# Supplementary material for: Structured tailored rehabilitation after hip fragility fracture: The ‘Stratify’ feasibility and pilot randomised controlled trial protocol
Source: PLoS One. 2024 Dec 17;19(12):e0306870. doi: 10.1371/journal.pone.0306870 (PMC11651604; doi:10.1371/journal.pone.0306870)
Supplement: S2 File — (PDF) [file pone.0306870.s002.pdf]

STUDY TITLE:

Structured Tailored Rehabilitation After Hip Fragility Fracture: The 'STRATIFY' Feasibility Randomised Controlled Trial

SHORT STUDY TITLE and ACRONYM

Rehabilitation after hip fracture: the Stratify-Hip trial.

Chief Investigator:

Dr Julie Whitney

Co-sponsored by

King's College London (KCL) and Guy's and St Thomas' NHS Foundation Trust

Funded by:

UKRI Future Leaders Fellowship [Grant Ref: MR/S032819/1].

Protocol version number and date:

V2.0, 31/01/2024

Name and address of Co-Investigator(s), Trial Manager and key study contacts.

Name:	Julie Whitney
Role:	Chief investigator

Name:	Katie Sheehan
Role:	Professor of Rehabilitation & Chief Scientific Officer

Name:	Catherine Sackley
Role:	Primary mentor

Name:	Salma Ayis
Role:	Statistician

Name:	Aicha Goubar
Role:	Statistician

Name:	Stefanny Guerra
Role:	Trial manager

Name:	Nadine Foster
Role:	Mentor

Name:	Ian D Cameron
Role:	Mentor

Name:	Emma Godfrey
Role:	Mentor

Name:	Celia Gregson
Role:	Mentor

Name:	Finbarr Martin
Role:	Mentor

Name:	Nicola Walsh
Role:	Mentor

Name:	Anna Ferguson Montague
Role:	PPI representative

PROTOCOL VERSION NUMBER AND DATE

Version Stage	Versions No	Version Date	Protocol updated & finalised by;	Detail the key protocol update

SIGNATURE PAGE

The Chief Investigator and the R&D (sponsor office) have reviewed this protocol. The investigators agree to perform the investigations and to abide by this protocol.

The investigator agrees to conduct the trial in compliance with the approved protocol, EU GCP, the UK Data Protection Act (2018), the Trust Information Governance Policy (or other local equivalent), the UK policy Framework for Health and Social Care research, the Sponsor's SOPs, and other regulatory requirements as amended.

Chief investigator

Julie Whitney

____

Signature Date

CONTENTS PAGE

SIGNATURE PAGE 5

CONTENTS PAGE 6

LIST OF ABBREVIATIONS AND DEFINITIONS 9

1 SUMMARY/SYNOPSIS 10

2 INTRODUCTION 11

3 PATIENT AND PUBLIC INVOLVEMENT (PPI) 12

4 TRIAL OBJECTIVES AND PURPOSE 13

5 STUDY DESIGN & FLOWCHART 14

5.1 Study Design 14

6 PARTICIPANT SELECTION 19

6.1 Participant inclusion criteria 19

6.2 Participant exclusion criteria 19

7 STUDY PROCEDURES 20

7.1 Timeline 20

7.2 Screening Procedures 20

7.3 Recruitment 20

7.3.1 Participants with capacity 20

7.3.2 Participants without capacity 21

7.3.3 Carers 21

7.3.4 Therapists 22

7.4 Randomisation procedures 22

7.5 Blinding 22

7.6 Schedule of assessments for each visit 22

7.7 Schedule of treatments for each visit 24

7.7.1 Low risk subgroup 25

7.7.2 Medium risk subgroup 28

7.7.3 High risk subgroup 32

7.8 Therapist training ... 35
7.9 Follow up procedures ... 36
8 MEDICAL EQUIPMENT (DEVICES) ... 36
8.1 Summary Description ... 36
8.2 Manufacturer ... 36
8.3 Model Details ... 36
8.4 Traceability ... 37
8.5 Intended Purpose ... 37
8.6 Detailed Description ... 37
8.7 User Training ... 37
9 END OF STUDY DEFINITION ... 38
10 ASSESSMENT OF SAFETY ... 38
10.1 Ethics Safety Reporting ... 39
10.2 Urgent Safety Measures ... 39
10.3 Trial Steering and Data Monitoring Committee ... 40
10.4 Ethics & Regulatory Approvals ... 40
11 COMPLIANCE AND WITHDRAWAL ... 40
11.1 Participant compliance ... 40
11.2 Withdrawal / dropout of participants ... 41
11.3 Protocol compliance ... 41
12 DATA ... 42
12.1 Data collection ... 42
12.2 Data handling and record keeping ... 43
12.3 Data sharing ... 44
12.4 Personal Data Breaches ... 44
13 MONITORING AND AUDITING ... 45
13.1 Stopping / discontinuation rules and breaking of randomisation code ... 45

13.2 Monitoring, quality control and assurance ... 45
14 STATISTICAL CONSIDERATIONS ... 46
14.1 Sample size ... 46
14.2 Analysis plan ... 47
14.3 Quantitative analysis ... 47
14.4 Progression criteria ... 47
15 PEER REVIEW ... 48
16 FINANCING ... 48
17 INSURANCE AND INDEMNITY ... 48
18 DATA CONTROLLER ... 48
19 REPORTING AND DISSEMINATION ... 49
20 APPENDICES ... 50
20.1 Appendix 1: SAE reporting flow diagram-non CTIMPs ... 50
20.2 Appendix 2: Information with regards to Safety Reporting in Clinical Investigations ... 51
20.3 Appendix 3: SADE Report Form – serious adverse device effect ... 54
21 REFERENCES ... 57

LIST OF ABBREVIATIONS AND DEFINITIONS

AE	Adverse Event
CI	Chief Investigator - The overall lead researcher for a research project. Chief investigators are responsible for the overall conduct of a research project.
CRF	Case Report Form
GSTFT	Guys and St Thomas NHS Foundation Trust
KCL	King’s College London
PI	Principal Investigator- An individual responsible for the conduct of the research at a research site.
Participant	An individual who takes part in a clinical trial
REC	Research Ethics Committee
R&D	Research & Development
SAE	Serious Adverse Event
Sponsor	The organisation or partnership that takes on overall responsibility for proportionate, effective arrangements being in place to set up, run and report a research project.
TMG	Trial Management Group
TSDMC	Trial Steering & Data Monitoring Committee

1 SUMMARY/SYNOPSIS

Title	Structured Tailored Rehabilitation After Hip Fragility Fracture:<br>The 'STRATIFY-HIP' Feasibility Randomised Controlled Trial
Protocol Short Title/Acronym	*Rehabilitation after hip fracture: the Stratify-Hip trial.*
IRAS Number	312631
REC Reference	
Study Duration	15 months
Health condition(s) or problem(s) studied	Hip fracture
Primary objective	*The primary objective of this feasibility and pilot randomised trial is to determine the treatment fidelity of the proposed intervention.*
Secondary objective (s)	*Secondary objectives seek to determine:*<br>*a) Count of screened, eligible, approach, recruited and retained participants and carers.*<br>*b) The acceptability of the intervention to participants, carers, and therapists.*<br>*c) Barriers and enablers to intervention delivery.*<br>*d) Acceptability, completeness, and descriptive comparison of outcome data collection.*<br>*e) Count of inadvertent unblinding of outcome assessors.*<br>*f) Count of adverse and serious adverse events.*<br>*g) Indicative sample size for a definitive trial.*
End of study definition	*The date when all data queries are resolved, and database locked which is anticipated to be month 15 from study start.*
Number of Participants	*60*
Study Type	*Feasibility randomised controlled trial*
Data collected/storage	Data will be collected on 1) a GSTFT hosted REDCap database accessible to the research team and GSTT therapists, 2) a KCL hosted SharePoint server accessible to the research team, and 3) paper-based data (consent forms, patient diaries, treatment logs prior to REDCap entry) will be stored in a site file at GSTFT and electronically transferred to KCL via encrypted emails. Site level and patient identifiable data from GSTFT will be Iron Mountain Archived at the end of the study. Data held by KCL will be archived at KCL.

2 INTRODUCTION

Each year, United Kingdom (UK) hospitals admit 70,000 men and women over the age of 60 years with hip fracture.[1] Even with surgery, 30% of patients die within a year.[2] Among survivors, 25% never walk again, and 22% transition from independent living to nursing homes.[2] This led 81 global societies to endorse a call to action to improve acute multidisciplinary care after hip fracture.[3]

A recent Cochrane systematic review supports rehabilitation in hospital as an effective approach to reduce mortality and adverse outcomes after hip fracture.[4] However, the nature of the rehabilitation interventions varied considerably limiting conclusions on the optimal components. This uncertainty has translated to NICE guidance being limited to daily mobilisation and regular physiotherapy review.[5]

The generalisability of the evidence is also limited as many rehabilitation trials attempted to account for differences in the hip fracture population by targeting homogenous subgroups such as patients with cognitive impairment,[6] women,[7] or from nursing homes.[8] It is therefore uncertain whether interventions deemed 'effective' are so for all patients, or for the targeted subgroup. This in turn poses challenges as to how these interventions may be implemented when subgroups compete for finite National Health Service (NHS) resources.

An intervention based on stratified rehabilitation may provide answers to these uncertainties. Stratified rehabilitation considers an entire population competing for resources to identify subgroups of patients with different risk of poor outcomes.[9] Subgroups are then matched to rehabilitation tailored to their needs to optimise outcomes across the entire population. Hip fracture survivors recently described this tailored approach as key to successful recovery.[10] Further, a stratified approach is regarded central to the progress of healthcare according to the NHS[9] and House of Lords Science and Technology Committee.[11] Although an approach of matching rehabilitation to patient subgroups with different risks of poor outcomes is intuitive and effective for other conditions, it has not been tested for rehabilitation after hip fracture.

A stratified approach to rehabilitation after hip fracture was subsequently developed. First, we developed and validated a subgrouping tool 'the stratify hip algorithm' (based on three multivariable prediction models) to identify patients at low-, intermediate- and high-risk of death and/or change in residence (to a higher level of care) using records for over 170,000 patients admitted to one of 173 hospitals in England and Wales.[12] The approach requires entry of 5 pieces of information to a website: https://stratifyhip.co.uk/ (age, sex, prefracture mobility, prefracture residence, and dementia diagnosis) to subgroup each patient.[12] Overall, 31% of patients were assigned low-risk, 28% medium-risk, and 41% high-risk across three outcomes (in-hospital death, change in residence, 30-day death).[12] Patients in the overall low-risk subgroup were typically less than 80 years old with the majority female (66%), admitted from home (91%), with outdoor mobility pre-fracture (83%), and no dementia diagnosis (95%). Compared to the overall low-risk subgroup, a greater proportion of patients in the overall medium-risk subgroup were older (94% aged 80 years or more), female (99%), and had a dementia diagnosis (14%). Compared to the overall medium-risk subgroup, patients in the high-risk subgroup were of a similar age (94% aged 80 years or more); however, a greater proportion was male (37%), with indoor/no mobility (69%), admitted from nursing/residential care (40%) with a dementia diagnosis (50%).

Next matched interventions were designed for each subgroup identified by the algorithm. These interventions were informed by qualitative interviews with patients,[13] physiotherapists,[14] and the multidisciplinary team,[15] an umbrella review of systematic reviews of older adults who underwent rehabilitation interventions in the acute setting,[16] recent systematic (including Cochrane) reviews,[4 17-20] international guidelines,[5 21-23] consultation with our public and patient involvement group 'TROOP' (further detail of TROOP available at www.ppitroop.co.uk), and an intervention development workshop and survey (following a nominal group technique[24]) to prioritize intervention components with key stakeholders.

The overarching purpose of a future main trial is to determine the clinical- and cost-effectiveness of adding a stratified care intervention to usual care designed to improve outcomes of acute rehabilitation for older adults after hip fracture. Several uncertainties will first be addressed through a randomised feasibility and pilot trial which this protocol details.

The primary objective of this feasibility and pilot randomised trial is to determine the treatment fidelity of the proposed intervention.

Secondary objectives seek to determine:

1. Count of screened, eligible, approached, recruited, and retained participants and carers.
2. The acceptability of the intervention to participants, carers, and therapists.
3. Barriers and enablers to intervention delivery.
4. Acceptability, completeness, and descriptive comparison of outcome data collection.
5. Count of inadvertent unblinding of outcome assessors.
6. Count of adverse events (AE) and serious adverse events (SAE).
7. Indicative sample size for a future definitive trial.

3 PATIENT AND PUBLIC INVOLVEMENT (PPI)

We involved patients and carers from proposal conception onwards. A 2-hour focus group of patients and carers at St. Thomas's Hospital informed the:

- target population as many carers expressed concern over disparities in the provision of rehabilitation based on patient characteristics such as the presence of dementia and/or admission from a residential/nursing care home (population selected – all patients surgically treated for hip fracture).
- outcomes for the multivariable models as all who took part expressed a goal of returning home as soon as possible (outcomes selected – time to death and time to change in residence).

In 2020, Sheehan supported the establishment of a PPI group which meets quarterly to discuss **T**rauma **R**ehabilitation (**O**rthopaedic) research for **O**lder **P**eople - '**TROOP**'. TROOP includes men and women from different ethnic backgrounds who reside at home across England. Members of TROOP contributed to the:

- intervention design during a workshop 06/22 and focus group 01/23. Members were given the opportunity to provide suggestions for the intervention and feedback on suggestions from others during a workshop and a focus group.
- development of educational materials to be provided as part of the intervention (focus group 04/23 and subsequent written feedback on materials via email).
- draft of the participant information leaflet and consent forms (written feedback via email).

We will continue our active collaboration with PPI members (currently eight members) of TROOP for the duration of the project. We will follow the UK Standards for Public Involvement to ensure this collaboration follows best practice and TROOP members continue to be reimbursed appropriately for all contributions.[25]

One PPI representative will take a leadership role for TROOP with respect to the current project and attend trial investigator group meetings. They will be sent materials (including an outline of the format) in advance of each meeting. They will be offered a pre-meeting with the lead applicant to discuss anything that is not clear.

TROOP members will also continue to meet quarterly. These meetings will include discussion of progress, interpretation of qualitative and quantitative results, and development and dissemination of plain English summaries of the project findings.

Finally, we will recruit a PPI member independent of TROOP for the trial steering committee through NIHR People in Research.

4 TRIAL OBJECTIVES AND PURPOSE

The **overarching purpose** of a future main trial is to determine the clinical- and cost-effectiveness of adding a stratified care intervention to usual care designed to improve outcomes of rehabilitation care for older adults after hip fracture. Several uncertainties will first be addressed through a randomised feasibility trial which this protocol details.

The **primary objective** of this feasibility and pilot randomised trial is to determine the treatment fidelity of the proposed intervention. **Secondary objectives** seek to determine:

1. Count of screened, eligible, approached, recruited, and retained participants and carers.
2. The acceptability of the intervention to participants, carers, and therapists.
3. Barriers and enablers to intervention delivery.
4. Acceptability, completeness, and descriptive comparison of patient reported outcome data collection.
5. Count of inadvertent unblinding of outcome assessors.
6. Count of AE and SAE.
7. Indicative sample size for a definitive trial.

5 STUDY DESIGN & FLOWCHART

5.1 Study Design

This is a feasibility parallel arm pragmatic single centre randomised controlled pilot trial with an embedded qualitative study.

The primary objective of this study (to determine the treatment fidelity of the proposed intervention.) will be met by analysis of data collected through written patient diaries and treatment logs after each intervention contact and observations during intervention delivery..

Secondary objectives of the study will be met by analysis of screened, eligible, approached, and randomised data, consent, and completion logs (secondary objective 1), analysis of assessments at intervention end and 12-weeks post-discharge (secondary objectives 4, 5, 7), and qualitative interviews of patients, carers and therapists at intervention end (secondary objectives 2, 3, 4). Further secondary objectives are to determine the count of AEs and SAEs identified through reporting procedures in place from the point of randomisation to 12-week follow-up.

A flow diagram providing a schematic overview of the study is provided in Figure 1. A study time/event matrix is available in Table 1.

Figure 1: Schematic overview of the study

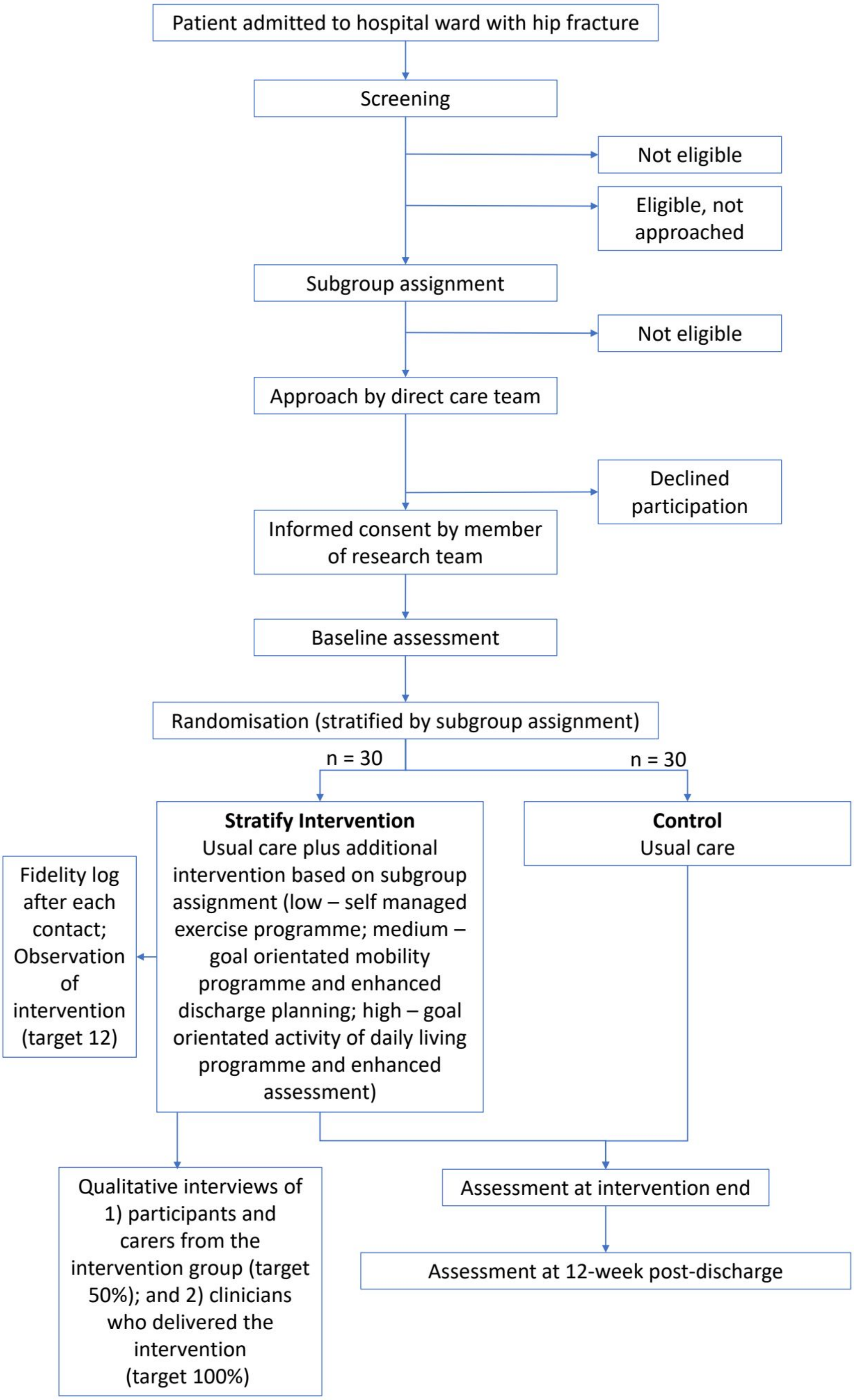

Table 1: Study time/event matrix.

Data	Form	Source	Completed by	Time point for collection					
				Recruitment	Baseline	Randomisation	Intervention (In hospital)	Intervention end	12-week follow-up
Screening log	Binary and Categorical	Patient notes	Site therapist	X					
Subgroup assignment log	Categorical	Patient notes	Site therapist	X					
Approach log	Binary and Categorical	Patient interview	Site therapist	X					
Contact details	Free text	Patient interview	Site therapist	X					
Consent log	Binary	Patient interview	Research team	X					
Age	Numerical	Patient interview	Site therapist		X				
Sex	Binary	Patient interview	Site therapist		X				
Ethnicity	Categorical	Patient interview	Site therapist		X				
Fracture type	Categorical	Patient notes	Site therapist		X				
Surgery type	Categorical	Patient notes	Site therapist		X				
Abbreviated Mental Test	Numerical	Patient notes	Site therapist		X				
Mini Nutritional Assessment	Numerical	Patient notes	Site therapist		X				
Hospital concerns about falls assessment	Numerical	Patient interview	Research team		X				
Residence	Categorical	Patient interview	Site therapist		X (prefracture)			X	X
Living status	Categorical	Patient interview	Site therapist		X (prefracture)			X	X
Mobility	Categorical	Patient interview	Site therapist		X (prefracture)				X
EuroQoL EQ-5D-5L	Numerical	Patient questionnaire	Research team		X			X	X
Barthel Index	Numerical	Patient questionnaire	Research team		X			X	X

Nottingham Extended Activities of Daily Living	Numerical	Patient questionnaire	Research team		X			X	X
Short Fallls Efficacy Scale-International	Numerical	Patient questionnaire	Research team		X			X	X
Numeric Rating Scale	Numerical	Patient questionnaire	Research team		X			X	X
New Mobility Score	Numerical	Patient questionnaire	Research team		X			X	X
Bespoke resource use form	Categorical	Patient questionnaire	Research team		X				X
Randomisation log	Binary	Computer generated randomisation	Research team			X			
Carer screening log	Binary and Categorical	Carer interview	Site therapist				X		
Carer approach log	Binary and Categorical	Carer interview	Site therapist				X		
Carer contact details	Free text	Carer interview	Site therapist				X		
Carer consent log	Binary and Categorical	Carer interview	Research team				X		
Carer age	Numerical	Carer interview	Research team				X		
Carer sex	Binary	Carer interview	Research team				X		
Carer education	Categorical	Carer interview	Research team				X		
Carer children	Binary	Carer interview	Research team				X		
Carer employment status	Categorical	Carer interview	Research team				X		
Carer relationship to participant	Categorical	Carer interview	Research team				X		
Treatment - control	-	-	Site therapist				X		

Treatment - intervention	-	-	Site therapist				X		
Treatment observations	Binary, categorical, and free text	Questionnaire	Research team				X		
Treatment logs	Categorical	Therapist questionnaire	Site therapist				X		
Deviation log	Free text	Therapist questionnaire	Site therapist				X		
Patient diary	Categorical	Patient diary	Patient/carer				X		
Length of stay	Numerical	Patient notes	Site therapist					X	
Mortality	Binary	Online death records	Research team					X	X
Readmission	Binary	Patient interview	Research team						X
Readmission diagnosis (as applicable)	Free text	Patient interview	Research team						X
Completion logs	Binary	Therapist questionnaire	Site therapist					X	X
Patient semi-structured interviews	Free text	Patient interview	Research team					X	
Carer semi-structured interviews	Free text	Patient interview	Research team					X	
Therapist semi-structured interviews	Free text	Therapist interview	Research team					X	

6 PARTICIPANT SELECTION

Participants will be recruited within 4 days of admission to a hospital ward at St Thomas's Hospital with hip fracture. In 2022, 255 patients were admitted to a hospital ward at St Thomas's Hospital with hip fracture. Previous trials of patients with hip fracture reported recruitment rates of 32% (of eligible and invited) in hospital.[26]

The recruitment target aims to have sufficient participants to provide the operational experience to plan a definitive trial; provide reasonably robust estimates of our feasibility outcomes; and to estimate the variability of the proposed patient outcomes to inform a future sample size calculation. A recruitment target of 60 participants (30 per treatment arm) will allow overall retention rate at 12-weeks to be estimated with precision of ±11%, using an exact 95% confidence interval, from previously observed retention rates of ~80% for the same population.[26] Assuming a non-differential retention rate of 80% at 12-week follow-up, this target will provide follow-up outcome data on ~24 participants per arm.

6.1 Participant inclusion criteria

We will include adults

- aged 60 years or more.
- admitted to hospital for surgical repair of a low energy hip fracture.
- who are willing and able to provide consent or assent depending on the level of cognitive impairment.

We sought to employ as inclusive eligibility criteria as possible to maximize representativeness of the population (and to ensure that the intervention is assessed for patients in all three risk subgroups). This decision followed our PPI consultations and findings from our systematic review detailing inequities in access to trials of rehabilitation after hip fracture surgery (27.3% of potential participants of 35 trials were excluded based on factors that stratify healthcare opportunities and outcomes).[27]

6.2 Participant exclusion criteria

We will exclude adults

- less than 60 years, to align with the National Hip Fracture Databases definition of the target population.[28]
- not surgically treated, as this treatment approach is reserved for around 2% of patients in the UK who are often at the end of life.[1]
- who broke their hip in hospital following admission for a different illness/injury as their anticipated care pathway and outcomes will vary from those who are admitted for hip fracture.
- who broke their hip following a high energy trauma e.g. road traffic accident.
- participating in other treatment trials and without agreement of both trial teams.

7 STUDY PROCEDURES

7.1 Timeline

The timeline for the trial is outlined in Table 1.

7.2 Screening Procedures

An anonymous screening log will document the number of adults admitted with hip fracture, the number screened (the number ineligible and reasons for ineligibility, and reasons for eligible but not screened), the number ineligible after subgroup assignment (we will target enrolment of 20 participants to each subgroup), the number approached, and the number who declined (and why). For those eligible but declined, we will also retain data on their subgroup assignment to determine whether the rates of decline vary by subgroup. The screening log will be completed by the clinical team who would routinely treat/access this cohort of patients to ensure the common law duty of confidentiality will be upheld. A site principal investigator (PI) who would routinely treat/access this cohort of patients will be responsible for ensuring the accuracy of the log.

7.3 Recruitment

Participants will be recruited within 4 days of admission to a hospital ward at St Thomas's Hospital with hip fracture. This is to allow sufficient time to deliver the intervention, given the average length of stay after hip fracture is 16 days.[29] Recruitment of this patient population within this timeframe has been completed successfully in previous UK trials.[30] Patients may be recruited pre- or post- operatively. In the rare instance where a patient is recruited pre-operatively but then does not go on to have surgery, they will be removed from the trial and replaced.

A member of the direct care team will decide whether a potential participant has capacity to give informed consent.

7.3.1 Participants with capacity

Potential participants will first be approached by a member of the direct care team who will determine their interest in taking part in the trial and gain consent to share their contact details with a member of the research team. Consent to contact will be documented in the CRF and medical notes. A member of the research team will subsequently provide an explanation of the aims, methods, benefits and potential harms and a participation information leaflet. Potential participants will be given at least 24 hours to consider their participation in the study. A member of the research team will subsequently answer any questions prior to obtaining written informed consent to enter and be randomised into the trial. Non-English language speaking older adults will be supported to consider their enrolment in the trial with the use of language support at the Trust.

7.3.2 Participants without capacity

For those who are considered as lacking capacity, agreement will be sought by a member of the research team from the patient and their consultee. If agreement is provided, the consultee will be provided with a consultee participant information leaflet and asked to sign a consultee declaration form. If the patient does not have a next of kin, the consultee may be a member of the patient's direct care team (who is not also a member of the research team). Subsequently, the member of the research team will seek assent from the patient for participation, as able. If a participant who lacks capacity indicates dissent, but their consultee advises involvement in the study, they will not be included in the study. This approach is in keeping with recommendations outlined by the Mental Capacity Act, 2005[31] and by the Alzheimer Europe Ethics of Dementia Research (http://www.alzheimer-europe.org/Ethics/Ethical-issues-inpractice/Ethics-of-dementia-research/Informed-consent-to-dementia-research).

Consultees will be invited to support the participant with data collection, or complete data collection on a participant's behalf. The number of consultees who declined to support data collection (and why) will be documented.

It is possible that a participant without capacity may see a capacity improvement during the study (e.g., for participants with pre/postoperative delirium that resolves). If this occurs, they will be informed of their enrolment in the study and what it involves, including the provision of the participant information leaflet and the opportunity to consent for themselves. They will be advised that they are free to withdraw from the trial at any point.

It is unlikely but possible a participant with capacity may see a capacity deterioration during the study. If this occurs, their original consent (with capacity) will be preserved. A consultee will be sought, provided a consultee participant information leaflet, and asked to sign a consultee declaration form. The consultee will be invited to support the participant with data collection, or complete data collection on a participant's behalf.

7.3.3 Carers

For all participants (irrespective of capacity status) enrolled in the intervention arm of the medium- or high- risk subgroups, their carer will be invited to join the intervention arm, if their carer is willing and able to provide consent.

A member of the direct care team will approach their carer in person or over the phone to tell them about the study and decide whether the carer has capacity to give informed consent. If so, the clinician will determine their interest in taking part in the trial and gain consent to share their contact details with a member of the research team. Screening, eligibility, approach, and consent to contact will be documented in the CRF. A member of the research team will subsequently provide an explanation of the aims, methods, benefits and potential harms and a carer information leaflet. Carers will be given at least 24 hours to consider their participation in the study. A member of the research team will subsequently answer any questions prior to obtaining written informed consent to enter the trial. The number of carers

who declined (and why) will be documented. Should a carer decline to take part, this will not affect the patient participant enrolled in the study.

7.3.4 Therapists

Therapists who were involved in delivery of the Stratify intervention arm of the feasibility trial will be invited to complete semi-structured interviews focused on treatment acceptability and fidelity (inclusive of barriers and facilitators to implementation). A member of the research team will outline the aims, methods, benefits and potential harms of the qualitative study and provide a participation information leaflet during therapist training prior to the start of the study. During this training sessions, therapists will be asked to provide consent to contact from the research team at the end of the study (their name and email address will be stored on an Excel Spreadsheet on secure KCL SharePoint server). At the end of the study a member of the research team will contact those who provided consent to contact and answer any questions prior to obtaining written informed consent to the interview study.

7.4 Randomisation procedures

The randomisation approach was designed with a statistician (SA) and validated for use prior to implementation. Once a participant has provided informed consent, they will be allocated a participant identification number (PID). Baseline data will subsequently be collected by the research team prior to randomisation. Randomisation will follow a 1:1 random allocation sequence stratified by subgroup assignment (low-, medium-, high- risk), using a secure internet-based system, developed and maintained by King's Clinical Trials Unit to ensure allocation concealment. Treatment allocation will be revealed (after randomisation) to a member of the research team, linked to the participant number, and the clinical team will be notified.

7.5 Blinding

The group allocator, assessor of adverse and serious adverse events, assessor of patient reported outcomes, and statistical analyst will be blind to group allocation.

7.6 Schedule of assessments for each visit

Participants will undergo screening, baseline assessment, assessment at intervention end, and assessment at 12-weeks post-randomisation. Assessments at baseline and intervention end will be in-person, over the telephone, or via MS TEAMS (the mode will be documented). Assessments at 12-weeks post-randomisation will be completed over the telephone or via MS TEAMS.

Screening for eligibility: Confirmation planned surgical intervention for hip fracture and aged 60 years or more. Additional assessment prior to consent will include a member of the direct care team's determination of capacity, confirmation whether the potential participant is enrolled in any other study, and subgroup assignment (to ensure no more than 12 participants from one subgroup enrolled to each arm of the study).

Baseline: Following consent and prior to randomisation, a member of the research team will collect the following participant characteristics: age, sex, ethnicity, fracture type, surgery type, Abbreviated Mental Test, Mini Nutritional Assessment, hospital concerns about falls

assessment, pre-fracture residential status (home, residential home, nursing home), living status (lives alone, with independent spouse, with dependent spouse, with family, with other), prefracture mobility. They will also collect patient-reported outcome measures which satisfy the core outcome set for hip fracture trials[32]:

a) health-related quality of life (EuroQoL EQ-5D-5L[33])
b) activities of daily living (Barthel Index;[34] Nottingham Extended Activities of Daily Living[35])
c) falls related self-efficacy (Short Falls Efficacy Scale-International[36 37])
d) pain (Numeric Rating Scale[39])
e) walking ability (New Mobility Score[40])

A bespoke resource use data collection form will also be collected. This form can be collected both prior to and/or after randomisation but will be completed prior to discharge (and intervention end).

<u>During the intervention:</u> The research team will conduct fidelity observations of at least twelve therapist-led sessions (four assessments for each subgroup in the intervention arm) following verbal consent from the therapist and participant engaged in each session. The research team will conduct fidelity observations of at least four carer training sessions (two assessments for medium- and high- subgroup in the intervention arm). Participants in the intervention arm will be asked to complete a participant diary indicating the extent to which they practices training without healthcare professionals.

<u>Intervention end</u>: At the intervention end, a member of the team will collect data on hospital length of stay, mortality (death records will be checked online prior to contacting the participant/their carer), place of residence (home, residential home, nursing home), living status (lives alone, with independent spouse, with dependent spouse, with family, with other), adverse/serious adverse events, as well as the following patient-reported outcome measures:

a) health-related quality of life (EuroQoL EQ-5D-5L[33])
b) activities of daily living (Barthel Index;[34] Nottingham Extended Activities of Daily Living[35])
c) falls related self-efficacy (Short Falls Efficacy Scale-International[36 37])
d) pain (Numeric Rating Scale[39])
e) walking ability (New Mobility Score[40])

They will also purposively sample participants with different risk subgroup assignment (low, medium, high), for telephone/MS TEAMS semi-structured interviews focused on treatment acceptability and fidelity (inclusive of barriers and facilitators to implementation). Interviews will target 50% of intervention participants but continue until no new themes are identified.[42]

At intervention end, purposively sampled therapists with different professional backgrounds (physiotherapist, occupational therapist, therapy assistant) and who were involved in the delivery of the Stratify intervention arm will be followed up by the research team by email to seek informed consent to participate in the qualitative study. Where consent is obtained, telephone/MS TEAMS semi-structured interviews focused on treatment acceptability and

fidelity (inclusive of barriers and facilitators to implementation) will be conducted. Interviews will target 100% of therapist involved in delivery of the intervention arm but continue until no new themes are identified.[42]

<u>12-week follow-up</u>: At 12-week follow up, a member of the research team will collect data related to mortality (death records will be checked online prior to contacting the participant/their carer), readmissions (and admitting diagnosis), place of residence (home, residential home, nursing home), and living status (lives alone, with independent spouse, with dependent spouse, with family, with other), any adverse/serious adverse events, as well as the following patient-reported outcome measures:

a) health-related quality of life (EuroQoL EQ-5D-5L[33])
b) activities of daily living (Barthel Index;[34] Nottingham Extended Activities of Daily Living[35])
c) falls related self-efficacy (Short Falls Efficacy Scale-International)
d) pain (Numeric Rating Scale[39])
e) walking ability (New Mobility Score[40])
f) bespoke resource use data collection form (including health and social care, informal care and paid/unpaid work)

Participants who are non-English language speakers will be supported to complete assessments (except patient reported outcome measures) with the use of language support at the Trust. Patient-reported outcome measures which have an established translated, validated, and (where applicable) culturally adapted version in the appropriate language will be circulated by post to the participant with a pre-paid envelope for return direct to the research team.

For carers recruited to the study, we will collect data relating to their age, sex, education, employment (not employed, part-time, full time), children (yes, no), and relationship to the participant. A member of the research team will purposively sample participants who are carers of those assigned to different risk subgroups (low, medium, high) for telephone/MS TEAMS semi-structured interviews focused on treatment acceptability and fidelity (inclusive of barriers and facilitators to implementation). Interviews will target 50% of intervention carers but continue until no new themes are identified.[42]

7.7 Schedule of treatments for each visit

Prior to randomisation, patients will be classified in one risk subgroup based on our stratification algorithm (comprising their age, sex, prefracture residence, prefracture ambulatory ability, and dementia status),[12] available at: https://stratifyhip.co.uk/.

Patients randomised to the control arm will receive usual physiotherapy and occupational therapy care. Usual care entails physiotherapy and occupational therapy from the day after surgery to the point of discharge, with a focus on discharge planning and sufficient recovery of activities of daily living and mobility for safe return to prefracture residence. Treatment logs will be used to record the components of usual care after each interaction with a treating therapist.

Patients randomised to the 'Stratify' intervention will receive usual care and an intervention based on their subgroup assignment. The intervention will start before the third

postoperative day and be delivered during the inpatient stay. Unless explicitly specified, intervention components will be delivered by a physiotherapist, occupational therapist, or therapy assistant (hereafter referred to as ‘therapist’) depending on staffing availability. Treatment logs will be used to record the components of usual care and the ‘Stratify’ intervention after each interaction with a treating therapist.

7.7.1 Low risk subgroup

Estimated 1hour 40minutes therapist time above usual care per participant

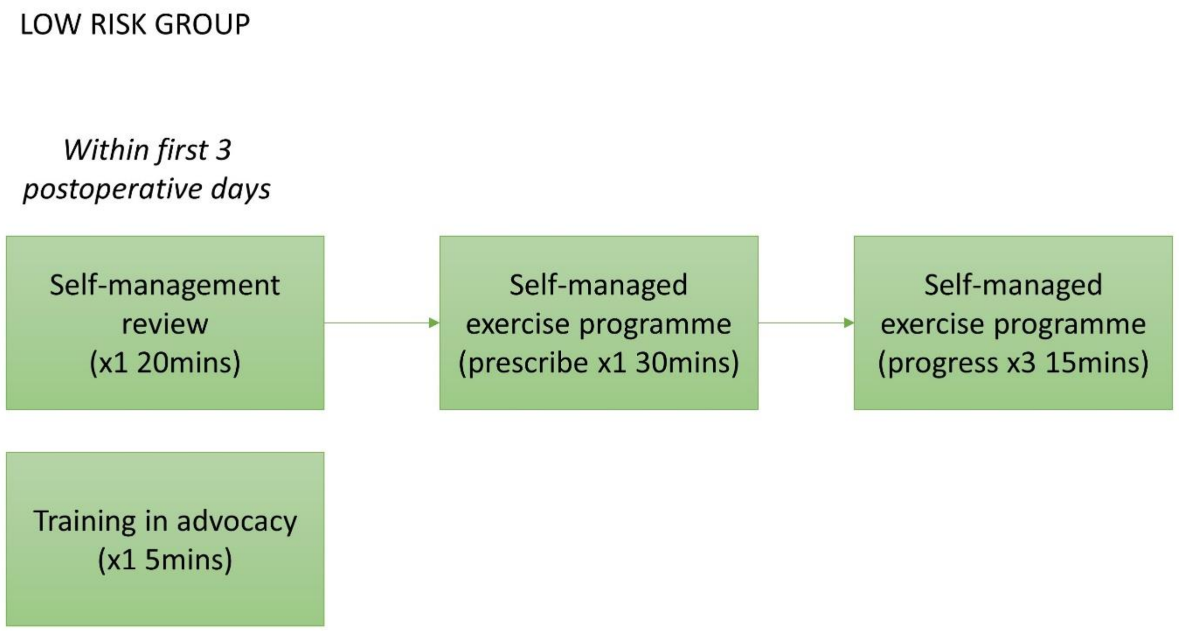

a) Self-management review

Assessment of barriers to self-management with the self-efficacy for managing chronic disease 6-item scale.[43] The participant should be made aware the ‘disease’ in this context refers to their hip fracture. Where a participant indicates a score of 5 or less the therapist should provide guidance to overcome identified barriers considering their capability, the opportunities, and their motivations.[44] This guidance for each question may include:

1. Fatigue:
    i. capability – encourage participants to plan to exercise and mobilise at the time of day where energy is higher. pacing – little and often.
    ii. opportunity - encourage participants to capitalise on visitors to support exercise/mobility as people who can e.g., find a chair should the participant become tired when mobilising.
    iii. motivation – specify the importance of aligning periods of less fatigue with periods they want to exercise and/or be mobile in pursuit of their recovery.
2. Pain:
    i. opportunity- outline what pain management the participant is entitled to.
    ii. capability – encourage participants to ask for pain management when they need it, and to refuse it when they do not need it.
    iii. motivation – specify the importance of aligning pain management with periods they want to exercise and/or be mobile in pursuit of their recovery.

3. Emotional distress:
    i. capability – provide social comparison as emotional support, emphasise positives of articulating concerns.
    ii. opportunity – encourage ongoing dialogue with friends, family, and healthcare providers; actively seek feedback. If welfare concerns, notify the direct care team and refer to appropriate specialties as needed (psychiatry, social care).
    iii. motivation –encourage participants to seek feedback from staff and visitors to provide emotional support for ongoing recovery.

4. Other symptoms/health problems:
    i. capability – encourage participants to continue to articulate any other symptoms/health problems they are experiencing to the direct care team.
    ii. opportunity- approach direct care team to address, with referral as needed.
    iii. motivation – encourage participants to seek support for other symptoms/health problems to prevent limiting ongoing recovery.

5. Need for (medical) support and/or 6. getting better by means other than medications: a participant may raise the need for support from therapies/nursing to mobilise/complete activities and exercise.
    i. capability – reassure participants of their capability for independent exercise and mobility following guidance from a therapist.
    ii. opportunity- highlight to participants the opportunity to exercise and mobilise independently in a safe environment with additional supervised support from therapists. Allow participant time to relay concerns about independent exercise/mobility, and discuss plans to overcome e.g., engage visitors.
    iii. motivation – encourage participants to inform staff and visitors of their exercise and mobility training as a source of motivation for their ongoing recovery.

b) Training in advocacy

Outline participants capability to complete activities of daily living, transfer, and mobilise independently during their hospital stay. Encourage them to do these activities for themselves, even if staff or visitors offer to do them, as the activities are a part of their rehabilitation and recovery. Discuss how it can be easier to accept help, but the motivation for declining is to ensure their ongoing recovery.

c) Self-managed exercise programme:

Progressive exercise programme inclusive of resistance and endurance training tailored to functional baseline based on programme based on a published exercise training programme and as specified in Box 1 below,[45] taught by a therapist who will populate a training plan template for the participant, which they can implement independently. Up to three additional sessions for monitoring and progression by a therapist.

Box 1: Progressive resistance and endurance training programme.

				Individually Tailored	
		**Type**	**Description**	**Start**	**Progression**
**Training once a day, on alternate days**	**0**	Warm up	Low intensity activity	Stepping on spot or walking.<br><br>Up to 5 minutes.	To 5 minutes if not achieved at programme start.
	**1**	Knee and hip extension	Squat	Mini squats in standing by wall/frame for balance support.<br><br>Focus on form. Erect spine, flex at hips, knee alignment, and forward gaze on descent. Control on ascent.<br><br>Target start of 3 sets of 10 repetitions with breaks between sets.	Sit to stand from raised seat. Avoid use of arm rests to stand or sit down.<br><br>Sit to stand from seat without raise. Avoid use of arm rests to stand or sit down.<br><br>Target 3 sets of 10 repetitions with breaks between sets for each progression.
	**2**	Hip abduction and extension	Unilateral hip abduction and extension	Standing unilateral hip abduction and extension by wall/frame for balance support.<br><br>Focus on form. Erect standing and controlled motion.<br><br>Target start of 2 sets of 10 repetitions on each side with breaks between sets.	Addition of TheraBand for resistance. Increasing from light resistance, to medium and heavy as able.<br><br>Target 2 sets of 10 repetitions on each side with breaks between sets for each progression.
	**3**	Ankle plantarflexion	Heel rise	Standing bilateral heel rise by wall/frame for balance support.<br><br>Focus on form. Erect standing and controlled motion.<br><br>Target start of 3 sets of 10 repetitions with breaks between sets.	Forefeet on 2cm support.<br><br>Forefeet on 4cm support.<br><br>Unilateral plantarflexion.<br><br>Target 3 sets of 10 repetitions with breaks between sets for bilateral training progression; Target 2 sets of 10 repetitions on each side with breaks between sets for unilateral training progression.

**Training daily**	**4**	Endurance	Walking	Safe baseline distance and assistance (mobility aid/person) determined by therapist.<br><br>Target start of 200m at low intensity with aids as needed. At least once daily.	Progression of distance, intensity, and assistance required.<br><br>Target progression to 600m at moderate intensity with aids as needed and/or more than once daily.

7.7.2 Medium risk subgroup

Anticipate 2 hours 15 minutes therapist time above usual care per participant.

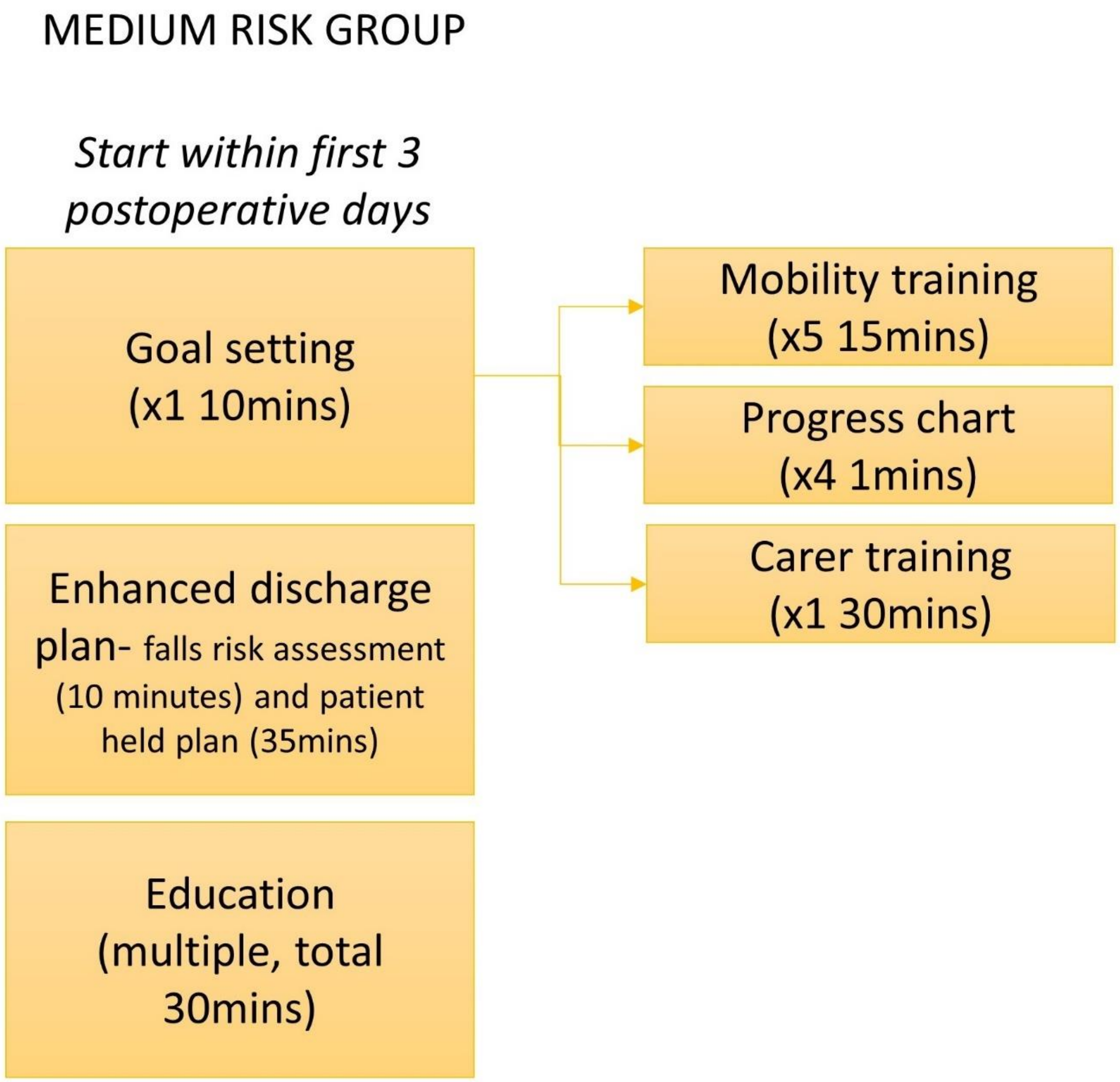

a) Education

1. Participants provided with information pack detailing what to expect from rehabilitation and their recovery.
2. Content discussed over first week (paced and reinforced) by therapist with participant and/or their carer (as available). Points to cover in discussion include:
    i. Getting better after surgery to fix a broken hip.
        - What does 'getting better' mean to the participant and/or carer. Discuss how rehabilitation is designed to support this.

    ii. Who is involved?
        - Focus on the participant, carer, and therapy team.

    iii. What happens in hospital?
        - Whether their pain is appropriately managed. If related to pain, discuss usual care process for requesting pain management prior to moving/ activities. Discuss any barriers to this request and how can rectify.
        - Whether they are feeling afraid of moving/activities due concerns about falls. If so, reinforce (regularly) that this is normal but that engaging in mobility/activities as able is the best thing they can do for themselves. Provide positive feedback on moving/activity achievements at regular intervals.
        - Whether the participant's ability to think or remember clearly is different since their operation. If concerns, explore and relay findings to multidisciplinary team.
        - Why it is important for them to do as much as they can for themselves safely in hospital. Discuss need to reinforce what is completed during supervised sessions in their own time (if therapist deems it safe to do so).
        - Discharge planning and whether the participant and/or carer feels they are involved in decision making. If not, why not, and how can rectify.

    iv. What happens after hospital?
        - Discuss likely care pathway including waiting times.
        - Discuss what they can be doing for themselves while they wait.

    v. What about later?
        - Manage expectations for the likely extent of therapy input once discharged from hospital and need to consider engagement with other services including charities to continue their recovery.

b) Goal-orientated mobility programme:

1. Collaborative (participant/carer and therapist) mobility goal setting. Encouragement of an ambitious 'outcome goal' to be achieved by the point of discharge. This 'outcome goal' should be accompanied by a series of intermediate 'behaviour goals' required to achieve the 'outcome goal'.

    Examples of domains for mobility outcome goals include (but are not limited to) walking in a busy indoor environment, walking outdoors, crossing a road. The goal set should seek to go beyond those set as part of usual care. The 'outcome

goal' may specify assistance in the form of equipment (crutches/frame) or people (See Box 2 for example).

Where possible/relevant, 'behaviour goals' should incorporate:

i. early supported mobility aid progression (from frame to rollator frame, crutches, or stick);
ii. early incorporation of dual task mobility (examples of cognitive dual task mobility: walking while reciting alphabet forwards/backwards, counting up in increments of 5, holding a conversation while continuing to mobilise; examples of motor dual task mobility: walking while carrying a small bag or empty cup, throwing and catching a ball while moving forward).

Box 2: Example of outcome goal and behaviour goal needed to achieve outcome goal.	
Outcome goal	I can walk outside on flat ground continuously for 3 minutes with the use of two crutches and accompanied by another person by the time I am leaving the hospital.
Behaviour goals	Walk continuously indoors on flat ground for 3 minutes with the use of two crutches independently.
	Walk indoors around a set of obstacles on flat ground while using two crutches independently.
	Walk indoors accompanied by another person and using two crutches while completing a series of cognitive tasks e.g., reciting every other letter of the alphabet, counting to 100 in increments of 5.

2. Up to five mobility training sessions with progressions (See Box 3 for example of progressions).

Box 3: Example of progressions towards behaviour goal	
Behaviour goal	Walk continuously indoors on flat ground for 3 minutes with the use of two crutches independently.
Progressions towards goal ↑	Walk continuously indoors on flat ground for 3 minutes with the use of two crutches accompanied by another person.
	Walk continuously indoors on flat ground for 3 minutes with the use of two crutches accompanied by another person.
	Walk continuously indoors on flat ground for 3 minutes with the use of a frame independently.
	Walk continuously indoors on flat ground for 3 minutes with the use of a frame accompanied by another person.

3. Progress chart template completed and placed at bedside indicating to the participant what they can complete independently and/or what they should be supported by team (including carer) to complete.

4. Carer (as available)
    i. training in the practical skills required to support safe practice of mobility towards a behaviour goal identified. The training will be delivered in person by a therapist on a one-to-one basis, last 30-minutes, and be tailored to the behaviour goals set. The carer will have the opportunity to support safe practice of mobility towards a behaviour goal in the presence of the therapist, who will answer any questions that may arise.
    ii. review of progress charts. A therapist will show the carer the participants progress chart which is placed at the participant's bedside (see '3' above). In particular, they will ensure the carer is able to identify behaviour goals which they
        - can practice with the participant when they visit and
        - should not practice with the participant when they visit.

    The therapist will also ensure the carer understands to approach a member of the therapy team if they are not sure how to support a behaviour goal, or if they do not feel comfortable to do so.

c) Therapist championed early enhanced discharge planning to include:

1. Template populated for a multifactorial falls risk assessment[46]. The template requires entry of data related to key domains of falls risk (falls history, gait, balance, mobility, strength, perceived functional ability, concerns about falls, visual impairment, cognitive impairment, urinary incontinence, home hazards, postural hypotension, and polypharmacy), the date of assessment (this may vary across domains), how they were assessed, the result, and any recommendation for teams in the postacute setting. Under 'how they were assessed?', for domains where a method of measurement is not suggested, populate with reference to the outcome measure/assessment used locally as part of usual care. Copies should be included in the medical chart and given to the team taking over the next stage of rehabilitation, and the participant and/or their carer (as applicable).
2. Discussion with participant and/or their carer to enable completion of participant held rehabilitation discharge plan template which includes six questions. Guidance on answering these questions:
    i. Waiting times for physiotherapy and occupational therapy (as required): early liaison with community teams to determine waiting times and likely offering on discharge home. If it is not standard practice for the participant to receive physiotherapy/ occupational therapy then this should be specified here, and they should be advised if they feel the need for either service to contact their GP to request a referral.
    ii. What I can work on after leaving the hospital: this is a discussion of behaviour goals which can continued to be practiced independently or with the support of a carer following discharge home. Discussion of appropriate self-progression as deemed safe for each individual participant.

iii. What else can I do to keep active after I leave the hospital: this is a discussion of activities completed prior to hip fracture and which activities may be safe to return to, and if so, how. For example, a participant who previously swam may wish to return to the pool. It would be good to guide their thinking on how they would get to the pool, get changed before and after, and to consider how they would get into and out of the pool (whether the pool has step entry or only a ladder). Consideration of private physiotherapy should finance permit. Summarise the discussion in the ‘my plan’ section.
iv. What upcoming appointments do I have: highlight to the participant that they can use this section to populate any appointments they are made aware of. Add details if a physiotherapy/occupational therapy home visit has been scheduled as part of early supported discharge.
v. What should I have with me: highlight they should keep their falls assessment and their discharge plan to hand to show to the therapist when they first visit.
vi. How do I contact people: provide contact details for physiotherapy and/or occupational therapy teams that provide support after discharge; here highlight that if in doubt, their GP is their point of contact.

Copies should be included in the medical chart and given to the team taking over the next stage of rehabilitation, and the participant and/or their carer (as applicable).

7.7.3 High risk subgroup

Anticipate up to 2 hours 45mins therapist time above usual care per participant.

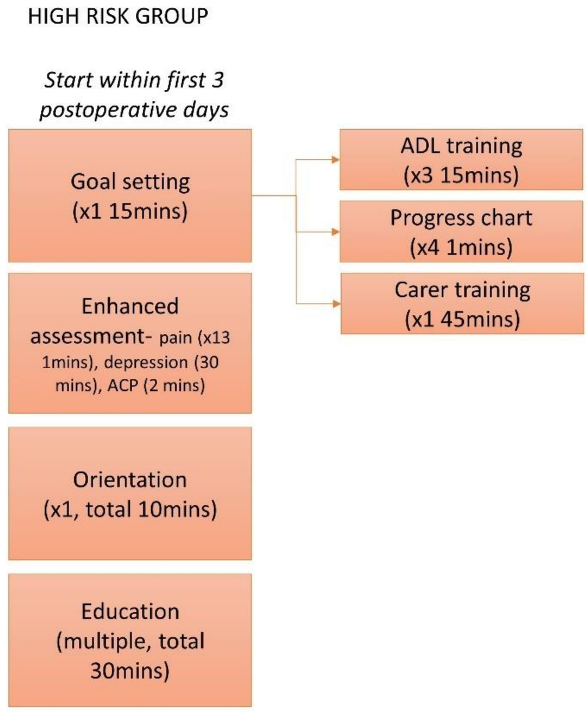

a) Education

1. Participants provided with information pack detailing what to expect from rehabilitation and their recovery.
2. Content discussed over first week (paced and reinforced) by therapist with participant and/or their carer (as available). Points to cover in discussion include:
   i. Getting better after surgery to fix a broken hip.
      - What does 'getting better' mean to the participant and/or carer. Discuss how rehabilitation is designed to support this.

   ii. Who is involved?
      - Focus on the participant, carer, and therapy team.

   iii. What happens in hospital?
      - Whether their pain is appropriately managed. If related to pain, discuss usual care process for requesting pain management prior to moving/ activities. Discuss any barriers to this request and how can rectify.
      - Whether they are feeling afraid of moving/activities due concerns about falls. If so, reinforce (regularly) that this is normal but that engaging in mobility/activities as able is the best thing they can do for themselves. Provide positive feedback on moving/activity achievements at regular intervals.
      - Whether the participant's ability to think or remember clearly is different since their operation. If concerns, explore and relay findings to multidisciplinary team.
      - Why it is important for them to do as much as they can for themselves safely in hospital. Discuss limited direct contact time with therapists and need to reinforce what is completed during supervised sessions in their own time (if therapist deems it safe to do so).
      - Discharge planning and whether the participant and/or carer feels they are involved in decision making. If not, why not, and how can rectify.

   iv. What happens after hospital?
      - Discuss likely care pathway including waiting times.
      - Discuss what they can be doing for themselves while they wait.

   v. What about later?
      - Manage expectations for the likely extent of therapy input once discharged from hospital and need to consider engagement with other services including charities to continue their recovery.

b) Orientation

Participants and/or carers (as available) requested by therapist to bring memoir materials such as framed photographs of family and friends, photo album, music device, reading materials, pillowcase/blanket, for bedside.

c) Enhanced assessment

1. Assessment of pain with the Algoplus dementia friendly pain scale from the first therapist contact and all contacts going forward.[47]
2. Assessment of depression by the therapist using the Cornell Scale for Depression in Dementia.[48] This should only be completed for participant s with no prior depression diagnosis. The scale has two parts - one to be responded by the participant's carer and the second by the participant themselves. The scales require responses based on 'the last week'. This should be completed following the first week in hospital. If the final score suggests a probable/definite major depression, notify multidisciplinary team, and suggest psychiatry referral.
3. The therapist will determine whether the participant has an advanced care plan (from medical chart/medical team). If there is no advanced care plan in place, refer to specialty who is responsible for supporting creation of an advanced care plan e.g. general practitioner following discharge
4. Prior to participant discharge. Ensure handover of pain history, depression assessment, and whether a new advance care plan was established, to community team.

d) Goal-orientated activities of daily living training programme

1. Collaborative (participant /carer and therapist) activity of daily living goal setting. Example of activity of daily living goal domains include (but are not limited to) bathing, grooming, dressing, continence, toilet use, transfers, mobility, stairs. It should be determined what 'outcome goal' is to be achieved by the point of discharge and what intermediate 'behaviour goals' are needed to achieve this goal (See Box 4 for example).

Box 4: Example of outcome goal and behaviour goal needed to achieve outcome goal.	
Outcome goal	With verbal direction and provision of a soaped sponge, I can wash myself in a seated shower chair by the time I am leaving the hospital.
Behaviour goals	In a seated position, reach down to touch toes following verbal direction
	In a seated position, lift left thigh without use of hands and hold for 5 seconds following verbal direction, repeat for right thigh
	In a seated position, lean forward and twist around to left to touch buttocks and hold for 5 seconds following verbal direction, repeat twisting to right
	Raise left arm and hold over head, repeat for right arm

2. Up to three activity of daily living training sessions with progressions and aids (as needed e.g., reaching aid, bed rail, raised toilet seat) (See Box 5 for example of progressions).

Box 5: Example of progressions towards behaviour goal	
Behaviour goal	In a seated position, lift left thigh without use of hands and hold for 5 seconds following verbal direction, repeat for right thigh
Progressions towards goal	In a seated position, lift left thigh and hold without use of hands for 3 seconds following verbal direction, repeat for right thigh
	In a seated position, lift left thigh and hold without use of hands for 1 seconds following verbal direction, repeat for right thigh
	In a seated position, lift left thigh and lower with control without use of hands following verbal direction, repeat for right thigh
	In a seated position, lift left thigh without use of hands following verbal direction, repeat for right thigh

3. Progress charts template completed and placed at bedside indicating to the participant what they can complete independently and/or what they should be supported by team (including carer) to complete.

4. Carers (as available)
   i. training in the practical skills required to support safe practice of behaviour goals identified. The training will be delivered in person by a therapist on a one-to-one basis, last 30-minutes, and be tailored to the behaviour goals set. The carer will have the opportunity to practice supporting safe training towards a behaviour goal in the presence of the therapist, who will answer any questions that may arise.
   ii. review of progress charts. A therapist will show the carer the participants progress chart which is placed at the participant's bedside (see '3' above). In particular, they will ensure the carer is able to identify behaviour goals which they
      - can practice with the participant when they visit and
      - should not practice with the participant when they visit.

   The therapist will also ensure the carer understands to approach a member of the therapy team if they are not sure how to support a behaviour goal, or if they do not feel comfortable to do so.

7.8 Therapist training

Therapists involved in the delivery of the stratified care intervention in the trial will attend an intervention training day organised by the research team. This will include: background to the study and study aims; study design; participant selection; study procedures (inclusive of protocol for approach prior to consent, introduction to the stratify-hip algorithm, subgroups, and matched interventions); and an explanation of the relevant study documentation inclusive of intervention treatment log, deviation log, AE and SAE reporting, and data collection. The training will also outline the aims, methods, benefits and potential harms of

the qualitative study and provide a participation information leaflet. Therapists will be asked to provide consent to contact from the research team at the end of the study.

Therapists involved in the delivery of usual physiotherapy and occupational therapy will attend an online training session on how to complete the usual care treatment log, AE and SAE reporting, and therapist data collection.

7.9 Follow up procedures

Participants and their carers (where applicable) will be followed-up by the research team via telephone/MS TEAMS at the end of the intervention and at 12-weeks post-randomisation to complete assessments and interviews. Therapists will be followed up by the research team by email at the end of the intervention to seek informed consent to participate in the qualitative study. Where consent is obtained, therapists will complete semi-structured interviews over the telephone/MS TEAMS with a member of the research team.

8 MEDICAL EQUIPMENT (DEVICES)

8.1 Summary Description

Prior to randomisation, patients will be allocated to a risk subgroup based on anonymous entry of data related to age, sex, prefracture residence, prefracture ambulatory ability, and dementia status. This allocation will be completed using the 'stratify-hip algorithm', a non-CE marked Class IIA medical device available at https://stratifyhip.co.uk/.[12] A backup version of the device is also available on an Excel spreadsheet. Patients randomised to the 'Stratify' intervention will receive usual care and an intervention based on their subgroup assignment as specified in section 7.8.

8.2 Manufacturer

The 'stratify-hip algorithm' was manufactured by a team led by Katie Sheehan at King's College London.

Name	Katie Sheehan
Address	2nd Floor Addison House<br>Guys Campus<br>Kings College London<br>SE1 1UL

8.3 Model Details

Name	Stratify-Hip
Number	1
Website version and date	Version 1; 31/07/2023
Excel version and date	Version 2305; 31/07/2023

8.4 Traceability

The same version of the 'stratify-hip algorithm' will be used for all participants enrolled in the trial.

8.5 Intended Purpose

The 'stratify-hip algorithm' was developed for information/reference only. The algorithm itself has not been modified since initial publication. In this feasibility study this algorithm is intended to be used as a prioritisation tool for additional rehabilitation, according to the protocol listed in section 7.8.

8.6 Detailed Description

The 'stratify-hip algorithm' is an algorithm available at https://stratifyhip.co.uk/.[12] We will combine this algorithm with a clinical protocol based on subgroup assignment. Therapists will navigate to the website. There they will enter five pieces of information related to a potential participant – age (5-year category), sex (male, female), prefracture ambulation (outdoor and indoor, indoor only, no ambulation), prefracture residence (home, nursing/residential care), dementia status (yes, no). Following entry of this information a 'combined outcome risk' for death and change in residence is generated. This will assign patients to a low, medium, or high risk subgroup. Potential participants who consent and are randomised to the intervention arm of the study will receive an intervention tailored to their subgroup assignment. The low risk subgroup will receive a self-managed exercise programme in addition to usual care. The medium risk subgroup will receive education, a goal-orientated mobility programme (with carer training as available), and early enhanced discharge planning. The high risk subgroup will receive education, enhanced assessment, orientation, and a goal-orientated activities of daily living programme (with carer training as available). See section 7.8 for further detail on the clinical protocol.

8.7 User Training

All therapists in the study will receive training in how to assign patients to a risk group using the website-based algorithm in advance of the study start. Therapists will be provided with guidance on

a) how to navigate to the website-based algorithm on a Trust computer
b) the data items required to complete the algorithm
c) how to select the appropriate variable level for each data item from the dropdown menus in the website-based algorithm
d) how to determine the risk assignment (the risk assignment is generated automatically once the variable levels have been selected from the dropdown menus)
e) how to use the Excel-based algorithm in the event they have any issues accessing the website

In addition, therapists supporting delivery of the intervention arm will be trained to deliver a matched clinical protocol based on the risk assignment from the algorithm in advance of the study start. Confirmation of attendance at the training session will be documented to ensure all therapists are trained in how to use the algorithm effectively and safely.

9 END OF STUDY DEFINITION

The end of the study is the date when the last participant has completed all assessments, all data queries are resolved, and database locked which is anticipated to be month 15 from study start.

10 ASSESSMENT OF SAFETY

Participant safety will be determined through the reporting of AE and SAE which will be coded in MedDRA. The period for AE reporting will be following the signing of the study consent form until final follow-up at 12-weeks.

An adverse event (AE) is defined as any untoward medical occurrence in a study participant, which does not necessarily have a causal relationship with the trial intervention. Adverse events that will be collected and reported in this trial are limited to:

1. an exacerbation of a pre-existing illness.
2. an increase in the frequency or intensity of a pre-existing episodic event or condition.
3. continuous persistent disease or a symptom present at baseline that worsens following administration of the trial intervention.

A serious adverse event (SAE) is defined as an untoward occurrence that:

1. results in death.
2. is life threatening (at the time of the event).
3. requires unplanned hospitalisation or prolongation of existing hospitalisation.
4. results in persistent or significant disability or incapacity.
5. Other ‘important medical events’ may also be considered serious if they jeopardise the participant or require an intervention to prevent one of the above consequences.
6. Results in a congenital anomaly or birth defect

NOTE: The term "life-threatening" in the definition of "serious" refers to an event in which the participant was at risk of death at the time of the event; it does not refer to an event which hypothetically might have caused death if it were more severe.

All deaths occurring from randomisation until final follow-up (12-weeks) or withdrawal from the study, irrespective of their relationship to the intervention, will be documented by a notification of death form and entered into the trial database within 24 hours of identifying the death. Cause of death will be recorded where available.

For the current intervention, prolongation of existing hospitalisation following a fall may be an expected SAE and related to the intervention, given the intervention encourages people to increase their exercise, mobility, and/or activities of daily living. A related and unexpected SAE is an event which is related to the intervention; and ‘unexpected’ – that is, the type of event is not listed in the protocol as an expected occurrence.

Where AEs/SAEs occur, the team (direct care team delivering the intervention and control arms) will follow Good Clinical Practice Guidelines for the reporting to the medical team responsible for the participant’s care and notify the Principal Investigator (PI). PIs at all sites will report all AEs/SAEs to the CI via the study electronic database (REDCap). The CI will review all AEs/SAEs against GSTFTs guide for assessment of whether the event is related

to the intervention or not. The CI will then be responsible for reporting SAEs to the Sponsors R&D office.

All SAEs that are to be reported to the R&D office will be signed and dated and completed by the CI. Where a related and expected SAE occurs that does not require immediate reporting, this SAE will be reported in the Annual Progress Report and copied to the R&D office, alongside any AEs that occur that are not classified as 'serious'.

Related and unexpected SAEs will be reported immediately upon knowledge of the event to the R&D office and always within 24 hours. Reports of related and unexpected SAEs will be submitted to the Main NHS/ HSC REC within 15 days of the Chief Investigator becoming aware of the event, using the appropriate template. The form will be completed in typescript and signed by the Chief Investigator. The main REC will acknowledge receipt of safety reports within 30 days. A copy of the SAE notification and acknowledgement receipt will also be sent to the R&D office. The SAE will also be documented as unexpected and related in the CRF, participants medical notes if they are in hospital at the time, annual report (with site copied), and on Datix.

A joint Trial Steering and Data Monitoring Committee (TSDMC) will be informed of the number, nature and review outcomes for all serious adverse events and be asked to recommend any necessary actions.

Where an SAE occurs that does not require immediate reporting, this SAE will be reported in the Annual Progress Report and copied to the R&D office, alongside any AEs that occur that are not classified as 'serious'. The final report will detail the number (events and individuals) and nature of all events (AEs and SAEs) reported to members of each site and/or research team and will be submitted to the REC with the sponsor copied.

Please see Appendix 1 and 2 for further information on reporting of SAEs.

10.1 Ethics Safety Reporting

The joint Trial Steering and Data Monitoring Committee (TSDMC) will be informed of the number, nature and review outcomes for all SAEs and be asked to recommend any necessary actions. Annual progress reports and a final report detailing the number (events and individuals) and nature of all events (AEs and SAEs) reported to members of the site and/or research team will be submitted to the REC with the sponsor copied.

Reports of related and unexpected SAEs (signed and dated by the CI) will be submitted to the Main REC within 15 days of the CI becoming aware of the event (with Sponsor copied), via the NRES template. The Main REC should acknowledge receipt of safety report within 30 days. A copy of the SAE notification and acknowledgement receipt will be submitted to Sponsor at GSTFT and stored in the TMF.

10.2 Urgent Safety Measures

A decision to implement an urgent safety measure (USM) can be made by the Sponsor, CI, PI and/or TSDMC in the event of identifying an immediate risk to participant safety. USM

identified shall take immediate effect and the event will be notified to the REC no later than 3 calendar days from the date the measures are taken. Any incident identified by a site that may result in an USM must be communicated to the CI immediately via the institutional study mailbox. If the CI and the Sponsor consider the USM to affect all participants, all PI must be informed of the USM. A protocol amendment will be submitted to the HRA and REC at the earliest opportunity (and within three days) following implementation of the USM. If the USM requires a temporary halt to the study, this will be notified by an amendment.

10.3 Trial Steering and Data Monitoring Committee

A joint TSDMC will be established prior to trial start to include the CI, trial manager, a sponsor representative, as well as an independent Chair, statistician, PPI representative, clinician, and health services researcher. At quarterly meetings, the Committee will provide advice, data monitoring, quality assurance, and safety monitoring. The committee may include open and closed sessions. Closed sessions will not be attended by the chief investigator or trial manager and may be used for data monitoring and/or other discussions at the discretion of the Chair.

10.4 Ethics & Regulatory Approvals

This trial was funded as part of a UKRI Future Leaders Fellowship (Sheehan PI), the application for which underwent external peer review. The trial was designed in collaboration with PPI members of TROOP (see Section 4.0) and TROOP members will have an active role in the management of the trial, as well as in the interpretation and dissemination of the trial findings.

The study requires regulatory approval from the following bodies: NHS REC and HRA Approval. Before any site can enroll patients into the study, the Chief Investigator/Principal Investigator or designee will ensure that the appropriate regulatory approvals have been issued, and NHS Confirmations of Capacity and Capability and Sponsor green lights are in place.

For any amendments to the study, the Chief Investigator, in agreement with the Sponsor, will submit information to the appropriate body for them to issue approval for the amendment. The Chief Investigator will work with R&D departments as well as the study delivery team to confirm ongoing Capacity and Capability for the study.

All correspondence with the Sponsor, REC and HRA will be retained. The Chief Investigator will notify the Sponsor and REC of the end of the study.

11 COMPLIANCE AND WITHDRAWAL

11.1 Participant compliance

Participants will be provided a diary where they will indicate compliance with unsupervised components of the intervention i.e., low risk subgroup independent progressive exercise training, medium-risk subgroup mobility training with carer, high-risk subgroup activity of daily living training with carer. Compliance with supervised components will be monitored through therapist treatment logs completed at the end of each intervention session.

At the end of the intervention, the research team will conduct semi-structured interviews with participants from the intervention arm (target 50%), and carers enrolled in the intervention arm (target 50%) to explore the acceptability of recruitment, randomisation, and the intervention, as well as fidelity of the intervention.

11.2 Withdrawal / dropout of participants

Participants are free to withdraw from the trial at any point or a participant can be withdrawn by their consultee or the CI/site PI. No sanctions will follow if the participant (or the consultee on their behalf) decides to leave the research at any time.

Participants (/consultees on the participants behalf) who wish to withdraw will have the option of withdrawal from:

- All aspects of the trial but continued use of data collected up to that point.
- All aspects of the intervention but continued use of data collected up to that point and continue with planned completion of questionnaires and interviews as well as collection of information from routine health records for the purpose of primary and some secondary outcomes.
- Any ongoing aspects of the trial that require patient contact or completion of questionnaires, but permission to collection of information from routine health records for the purpose of primary and some secondary outcomes.

Participants can be withdrawn by the CI/site PI following:

- A change in the health status of the participant that the clinical team report justifies withdrawal.

For participants who die or who are not responsive to attempts to contact for assessments and interviews at intervention end and 12-week follow-up, they will be withdrawn from the trial but the research team will continue to use data collected up to that point, and collect information from routine health records for the purpose of some secondary outcomes. If withdrawal occurs, the primary reason for withdrawal will be documented in the participant's case report form (CRF) (as available). Participants who withdraw will not be replaced.

11.3 Protocol compliance

A protocol deviation is defined as an unintended departure from the expected conduct of the study protocol, which does not need to be reported to the sponsor. The CI will monitor protocol deviations by monitoring treatment logs completed by treating therapists after each session against the treatment allocation. Any deviations noted will be listed in a deviation log. Significant deviations to the protocol or deviations which are found to frequently recur will be assessed by the CI to see if an amendment to the protocol is required. Any significant deviations to the protocol or recurring deviations will be reported to sponsor and action taken through Corrective and Preventative Actions.

A 'serious breach' is defined as a breach likely to effect to a significant degree:

- The safety or physical or mental integrity of the participants of the trial;
- The scientific value of the trial.

The CI and sponsor will be notified by the site PI immediately if a serious breach occurs. The breach will then be reported to the REC Committee with the sponsor in copy within 7 calendar days of the breach being confirmed as serious.

12 DATA

12.1 Data collection

Data collection, including who the data are to be collected by, from where, the data type and the time point for collection, is outlined in Table 2. Data collection at baseline and intervention end will be in-person or over the telephone/MS TEAMS (the mode will be documented). Assessments at 12-weeks post-randomisation will be completed over the telephone or MS TEAMS.

The following participant characteristics will be collected to enable baseline comparison: age, sex, fracture type, surgery type, Abbreviated Mental Test, Mini Nutritional Assessment, hospital concerns about falls, prefracture residential status, living status, prefracture mobility. The acceptability, completeness, and descriptive comparison of patient reported outcome data collection (secondary objective 4) will be assessed through collection of patient-reported outcome measures (via standardised tools) which satisfy the core outcome set for hip fracture trials.[32] Additional patient-reported outcome measures (via standardised tools) which capture putative mechanisms (falls related self-efficacy, walking ability) for an effect on health-related quality of life (as the proposed outcome for a definitive trial) will also be collected.

Additional quantitative outcomes captured will include length of stay, death, readmissions, place of residence and living status (to determine if change to higher level of care, including informal care (e.g., living status change from alone to with family)) as key performance indicators of care after hip fracture surgery; and resource use using a bespoke data collection form to assess feasibility of collecting health economic data while minimising the burden on patients.

For patients who do not speak English, patient-reported outcome measures which have an established translated, validated, and (where applicable) culturally adapted version in the appropriate language will be circulated by post to the participant with a pre-paid envelope for return. Patient characteristics and additional quantitative outcomes will be collected using language support at the Trust.

For carers recruited to the study, we will collect data relating to their age, sex, employment (not employed, part-time, full time), children (yes, no), and relationship to the participant.

For therapist who provide consent to contact during therapist training, their name, profession, and email addressed will be collected directly in an Excel spreadsheet stored on a secure KCL SharePoint server.

We will assess treatment fidelity through analysis of treatment logs completed by therapists following each therapist supervised session, observations of intervention delivery and patient diaries completed following each unsupervised session. Finally, we will capture qualitative data on acceptability, and perceived barriers and facilitators to intervention fidelity (considering treatment design, training of providers, delivery, receipt, and enactment) through transcribed semi-structured interviews with participants (target 50%), carers (target 50%) from the intervention arm, and therapists (target 100%) at the end of the intervention.

12.2 Data handling and record keeping

Identifiable data will be collected in REDCap hosted by the Trust (GSTFT) or a password protected file on a secure KCL SharePoint server for those who provide informed consent. REDCap is a secure browser-based, metadata-driven EDC software and workflow methodology for designing clinical and translational research databases. This database will specify the participant identification number. Password protected user entry (to REDCap/SharePoint) will be limited to therapists and members of the research team who need to contact participants/consultees/carers to complete telephone/MS TEAMS assessments, interviews, and/or circulate information including carer information leaflets and/or end of study summary. This identifiable data will be held separately to the research data.

REDCap will be used for additional pseudo-anonymised data collection on two additional databases. These additional databases will be pseudo-anonymised with the use of the patient identification number as the primary identifier of the participant for data entered. Password protected user entry will be limited to therapists and members of the research team who need to enter or analyse data. The databases will be programmed to generate notifications for possible data entry errors and missing key data points.

- The first database will include anonymous data related to screening, eligibility, and approach data for both participants and carers (where applicable), randomisation, and allocation, and treatment logs entered by therapists, and observations entered by a member of the research team. All data will be accessed by authorized members of the research team for analysis, users will not be blind to treatment allocation.
- The second database will include participant characteristics collected at baseline; patient reported outcome measures at baseline, intervention end and 12-week follow-up; resource use at baseline and 12-week follow-up; length of stay at intervention end; readmissions at 12-week follow-up; and place of residence and living status at intervention end and follow-up. All data will be entered and accessed by authorized members of the research team for analysis, users will be blind to treatment allocation.

Pseudo-anonymised patient diaries will be shared with KCL to enable an assessment of fidelity of unsupervised intervention components by the research team. These paper records will be stored in a locked cabinet in a secure room at GSTFT with monthly electronic transfer from GSTFT to KCL where they will be stored on a secure KCL SharePoint server. For patients who do not speak English, pseudo-anonymised patient-reported outcome measures returned by post will be stored in a locked cabinet in a restricted access room and building at KCL.
At the end of the intervention, audio-recordings of semi-structured interviews will be collected from participants, carers and therapists by the research team. These audio-recordings will be

uploaded by the research team to a secure KCL SharePoint server accessible only to the research team, labeled with the participant identification number after which the recording will be deleted from the audio recording device. Recordings will be transcribed verbatim by an external transcription company with appropriate service level agreement and anonymised after which the audio-files will be permanently deleted.

After completion of the trial, site level and patient identifiable data (including consent forms) will be archived using Iron Mountain Archiving at an offsite external archiving unit for 5 years after which it will be destroyed in line with their policies on data destruction. Following publication of the primary paper, anonymised electronic REDCap data will be exported and stored alongside anonymised transcriptions of interviews on the King's Open Research Data System (https://www.kcl.ac.uk/researchsupport/managing/preserve), with proof of ethical approval as a condition of access. The data will be checked by the information governance team at KCL to confirm anonymity prior to storage on the King's Open Research Data System. For patients who do not speak English, pseudo-anonymised patient-reported outcome measures returned by post will be stored in a locked cabinet in a restricted access room and building at KCL for 5 years after which they will be destroyed in line with KCL policies on data destruction.

12.3 Data sharing

Data will be shared between GSTFT and KCL in adherence with the Data Protection Act 2018. A data transfer agreement will be established to allow for transfer of data collected by therapists at GSTFT to the research team at KCL. Therapists at GSTFT involved in recruitment and intervention delivery are responsible for the collection of this data, its recording and quality. Participants will be required to consent to this data transfer prior to enrollment in the trial.

Identifiable data will be collected in REDCap hosted by the Trust (GSTFT) or a password protected file on a secure KCL SharePoint server for those who provide informed consent. The database will be accessible by the therapists at GSTFT and authorized members of the research team to arrange telephone assessments, telephone/MS TEAMS interviews, to send the trial results by post (if a participant selects this option), and to send patient-reported outcome measures by post for participants who are non-English language speakers, this database will specify the participant identification number.

Pseudo-anonymised screening, eligibility, approach, randomisation and allocation, and treatment logs will be entered by therapists to a REDCap database. The database will be pseudo-anonymised with the use of the patient identification number as the primary identifier of the participant for data entered. The database will be accessible by the therapists at GSTFT and authorized members of the research team at KCL for analysis.

12.4 Personal Data Breaches

Personal data breaches are defined as a security incident that has affected the confidentiality, integrity, or availability of personal data. In short, there will be a personal data breach whenever any personal data is lost, incorrectly destroyed, corrupted, or disclosed; if someone accesses the data or passes it on without proper authorization; or if the data is made unavailable, for example, when it has been encrypted by ransomware, or accidentally lost or destroyed.

Personal data breaches will be immediately reported to the CI, Sponsor's, and to the Data Protection Officer/Information Governance Department of the site that incurred the breach. The report will include full details as to the nature of the breach, an indication as to the volume of material involved, and the sensitivity of the breach (and any timeframes that apply), steps that have been taken to mitigate the risk (trying to retrieve the data asking third parties to delete information that was sent to them in error) to enable an assessment of the full risk/impact of the breach. The Sponsor will determine whether the breach meets the definition of a serious breach and warrants reporting to the regulators including the ICO https://ico.org.uk/for-organisations/report-a-breach/personal-data-breach-assessment/.

Sites will additionally follow their Trust incident reporting mechanisms and will document this within their trial master file in the form of a file note provided by the sponsor with Corrective and Preventative Actions addressed.

13 MONITORING AND AUDITING

The Chief Investigator will be responsible for the ongoing management of the study. The Sponsor will monitor and conduct audits on a selection of studies in its clinical research portfolio. Monitoring and auditing will be conducted in accordance with the UK Policy Framework for Health and Social Care and in accordance with the Sponsor's monitoring and audit procedures.

13.1 Stopping / discontinuation rules and breaking of randomisation code

The trial completion is the date when all data queries are resolved, and database locked which is anticipated to be month 15 from study start. There are no criteria for premature discontinuation of the trial (as a feasibility trial). There are no anticipated circumstances under which the randomisation codes may need to be broken.

13.2 Monitoring, quality control and assurance

The trial will be conducted in compliance with the approved protocol, the Declaration of Helsinki (2008), the principles of Good Clinical Practice (GCP) as laid down by the Commission Directive 2005/28/EC with implementation in national legislation in the UK by Statutory Instrument 2004/1031 and subsequent amendments, the UK Data Protection Act, and the UK Policy Framework for Health and Social Care Research, the Mental Capacity Act 2005, and other national and local applicable regulations.

The trial will be led and managed by the CI with the support of the trial manager. This will include co-ordination of all day-to-day aspects of the trial from initial set-up to closeout, including: development of the systems, protocol and other essential documents; REC/HRA submission and amendments; set-up and management of trial management group (TMG) and TSDMC meetings; planning and provision of site initiation visit; day-to-day liaison with site; study monitoring oversight; safety management and reporting, progress reporting to REC, TSDMC, Sponsor and funder; performance of site closure at end of study; provision of end of study notifications to HRA and REC; preparation of files for archiving.

The CI will chair the TMG, which will also include the investigators, PPI representative, trial manager, and trial statisticians meeting at least bi-monthly throughout the trial and more frequently during the earlier phases. The TMG will closely monitor study progress, identifying and addressing practical, scientific, and financial issues as they arise. The TMG will report progress to the Sponsor and the independent TSDMC.

The TSDMC will include the CI and trial manager, as well as an independent Chair, statistician, PPI representative, clinicians, and health services researchers. The committee will provide advice, data monitoring, quality assurance, and safety monitoring. The committee may include open and closed sessions. Closed sessions will not be attended by the chief investigator or trial manager and may be used for data monitoring and/or other discussions at the discretion of the Chair.

The research team will monitor treatment logs completed by treating therapists after each session against the treatment allocation and explore any quality control issues with treating therapists. Additionally, any quality control issues identified during treatment observations will be explored with the treating therapists.

The trial database will be programmed to generate notifications for possible data entry errors and missing key data points.

Prior to publication, the presentation of results (text, tables and figures) will be reviewed by the trial management group to ensure the anonymity of participants will be preserved. In particular, we will review variable level counts and identify any which are small and in combination with other data could render information potentially identifiable. If this occurs, we will categorize the variable further to ensure anonymity is preserved. If further categorization is not possible, we will suppress the potentially identifiable data and report the extent to which data suppression was employed in the results.

14 STATISTICAL CONSIDERATIONS

14.1 Sample size

The sample size was determined in consultation with the trial statistician. The recruitment target of 60 participants aims to have sufficient participants to provide the operational experience to plan a definitive trial; provide reasonably robust estimates of our feasibility outcomes; and to estimate the variability of the proposed patient outcomes to inform a future sample size calculation. A recruitment target of 60 participants (30 per treatment arm) will allow overall retention rate at 12-weeks to be estimated with precision of ±11%, using an exact 95% confidence interval, from previously observed retention rates of ~80% for the same population.[26] Assuming a non-differential retention rate of 80% at 12-week follow-up, this target will provide follow-up outcome data on ~24 participants per arm.

This required sample size will be attainable in practice. In 2022, 255 patients were admitted to a hospital ward at St Thomas's Hospital with hip fracture. Previous trials of patients with hip fracture reported recruitment rates of 32% in hospital.[26]

14.2 Analysis plan

A statistical analysis plan will be finalised ahead of database locking and reporting will follow the CONSORT guidance for pilot and feasibility studies.[49] All analysis will select participants according to the principles of intention to treat.[50]

14.3 Quantitative analysis

A CONSORT flow diagram will display data specifying counts of screened, eligible, approached, randomised, and completed enabling estimation of eligibility, recruitment, consent and follow-up rates.[49] Confidence intervals for recruitment and retention rates will be produced to inform assumptions for planning the definitive trial. Rates of screened, eligible, approach and recruited will also be estimated for carers of participants enrolled into the medium- or high-risk intervention arm. Completion rates will be estimated for outcome measures collected at each time-point. Baseline characteristics will be summarised by allocated arm (and by subgroup assignment) with descriptive statistics (measures of central tendency and dispersion) to enable assessment of baseline comparability of arms (a degree of imbalance is anticipated in this small feasibility study). Patient-reported outcomes and treatment fidelity will be summarised by allocated arm (and by subgroup assignment) at each follow-up, with descriptive statistics (measures of central tendency and dispersion). Between-arm differences, including in changes from baseline, will be reported for the patient-reported outcomes with corresponding measures of dispersion to enable an assessment of sensitivity to change to inform primary outcome selection for a definitive trial and a formal power calculation for this outcome for a definitive trial.

For patients who do not speak English, the count of pseudo-anonymised patient-reported outcome measures circulated and returned by post will be documented. The content of the patient-reported outcome measure will be described narratively ensuring participant anonymity is preserved (the language versions circulated will not be specified in reporting).

The quantitative analysis will be completed by a trial statistician using R (https://www.r-project.org/) (or other well validated statistical packages) after database lock at trial end. The TSDMC will monitor screening/eligibility, approach and randomisation rates and safety reporting. There are no additional interim analyses planned. Qualitative analysis

Qualitative data transcribed verbatim from semi-structured interviews and analysed using a thematic analysis approach.[51] The analysis will follow a deductive approach informed by the categories of treatment fidelity (design, training of providers, delivery, receipt and enactment), and to identify barriers and/or facilitators (inclusive of acceptability) to future implementation.[52]

The intervention observations will be sampled against therapists logs to further assess fidelity. Free text entries made during observations will be summarised narratively.

14.4 Progression criteria

To mitigate the risk of ongoing uncertainty at the end of the feasibility trial, we propose progression criteria outlined in Figure 2.[53]

Figure 2: Progression criteria

	GO	AMEND	STOP
**Recruitment**	≥40% eligible	21-39% eligible	≤20% eligible
**Recruitment**	≥50% eligible recruited	31-49% eligible recruited	≤30% eligible recruited
**Randomisation**	≥70% of those recruited randomised	49-70% of those recruited randomised	≤48% of those recruited randomised
**Fidelity**	≥80% sessions included all intervention components as described	51-79% sessions included all intervention components as described	≤50% sessions included all intervention components as described
**Outcome, 12-weeks**	≥80% completeness of EQ5D at 12-week follow-up	51-79% completeness of EQ5D at 12-week follow-up	≤50% completeness of EQ5D at 12-week follow-up

15 PEER REVIEW

This study was peer reviewed prior to the funding award by UKRI (Future Leaders Fellowship scheme).

16 FINANCING

This study is supported by a UKRI Future Leaders Fellowship [Grant Ref: MR/S032819/1]. The award offer was made on the 16th September 2019. The funding will be in place to the end of February 2025, with the possibility of a subsequent 3-year extension (subject to peer review).

17 INSURANCE AND INDEMNITY

This study is co-sponsored by King's College London (KCL) and Guys and St Thomas' NHS Foundation Trust (GSTFT). The co-sponsors will, at all times, maintain adequate insurance for the design, management and conduct of the study: (a) KCL through its' own professional indemnity (Clinical Trials) & no-fault compensation policy; and (b) GSTFT through NHS Resolution cover, in respect of any claims arising as a result of negligence by its employees, brought by or on behalf of a study participant.

18 DATA CONTROLLER

Guy's and St Thomas' NHS Foundation Trust (GSTFT) and King's College London (KCL) are co-sponsors of this research project and have shared Data Controller responsibilities. Where Personal Data is disclosed by GSTFT to KCL or vice versa, directly or indirectly to satisfy the requirements of the Protocol, or for the purpose of monitoring or reporting AE/SAE, or in relation to a claim or proceeding brought by a Participant in connection with the Trial, KCL and GSTFT agree to comply with the obligations placed on a Controller by the Data Protection Legislation. This is not limited to, but includes, being

responsible for and able to demonstrate compliance with the principles relating to Processing of Personal Data (Article 5 UK GDPR).

GSTFT and KCL have outlined their Data Controller to Controller arrangements in an overarching Master Data Sharing Agreement which sets out the principles of data sharing in accordance with UK GDPR, regulatory and statutory laws. GSTFT and KCL have agreed to the mutual study specific Joint Controller data sharing template which details their individual roles and responsibilities at a study level.

19 REPORTING AND DISSEMINATION

The study protocol will be made available on clinicaltrials.gov.

The results of the study will be summarised in plain English and made available on the teams public and patient involvement group webpage (www.ppitroop.co.uk) and Twitter page (@TROOP_PPI) as well as via the Royal Osteoporosis Society's Bone Matters e-newsletter (circulations in excess of 20,000). Participants will be offered the option of having the plain English summary posted directly to them during the consent process.

The results of the study will be published in open-access peer reviewed journals. The findings will be presented at national conferences (British Geriatrics Society; British Orthopaedic Society) and international conferences (Fragility Fracture Network (FFN)).

We will also disseminate results through the European Geriatric Medicine Society (past president Martin), Chartered Society of Physiotherapy (members Sheehan, Sackley); Royal College of Occupational Therapists (Fellow, Sackley); Royal National Osteoporosis Society (guideline committee, Gregson); and the FFN Hip Fracture Recovery Research Group (Chair, Sheehan).

Following publication of the primary paper, an anonymised dataset (anonymity confirmed by the information governance team at KCL) will be preserved indefinitely on the King's Open Research Data System (https://www.kcl.ac.uk/researchsupport/managing/preserve), with proof of ethical approval as a condition of access.

20 APPENDICES

20.1 Appendix 1: SAE reporting flow diagram-non CTIMPs

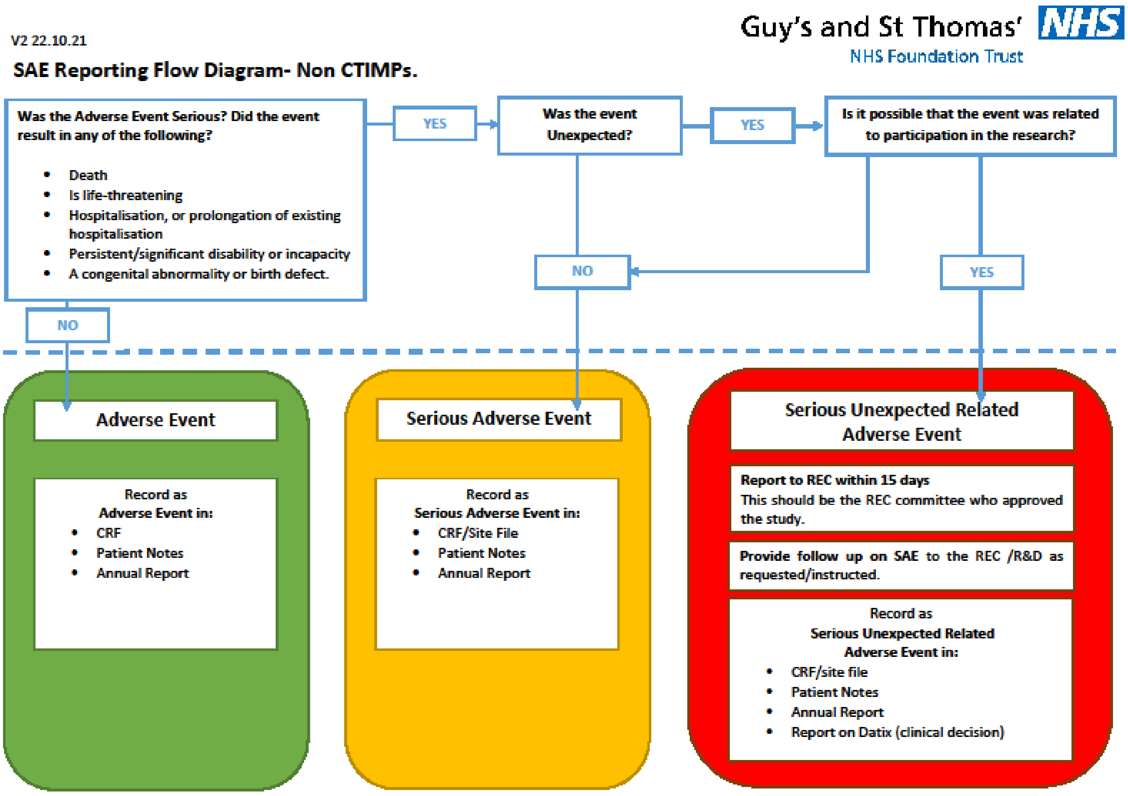

20.2 *Appendix 2: Information with regards to Safety Reporting in Clinical Investigations*

Term	Definition	Reporter	Reported to	Reporting Timeline from awareness of the event
Adverse Events that affect the device operators	Including injuries to staff from use of the device.	Investigator	Sponsor	As agreed with sponsor. (Preferably immediately, no later than three calendar days)
Adverse Device Effect (ADE)	Adverse event related to the device. Including resulting from the instructions, deployment, implantation, installation, the operation, any malfunction, user error or intentional abnormal use.	Investigator	Sponsor/ Manufacturer	As agreed with sponsor. (Preferably immediately, no later than three calendar days)
Serious Adverse Device Effect (SADE)	SAE related to the device	Investigator	Sponsor	Immediately, no more than 24hrs of becoming aware of the event
		Investigator/ Sponsor	MHRA	Within 2 calendar days of becoming aware of the event. Updates within 7 calendar day.
		Investigator/ Sponsor	MHRA Adverse Incident Centre	On CE or UKCA marked devices
Unanticipated Serious Adverse Device Effect (USADE)	Unanticipated SADE related to the device. Anticipated SAEs	Investigator	Sponsor	Immediately, no more than 24 hours

	(ASADE) can also be listed in the protocol.	CI/ Sponsor	MHRA/ REC	Within 15 calendar days of the CI becoming aware of the event
Device Deficiency (DD)	Inadequacy of device related to its identity, quality, durability, reliability, safety or performance. This may include malfunctions user error or inadequacy of information form manufacturer.	Investigator or Sponsor	Sponsor<br><br>MHRA	Immediately, no more than 24 hours of becoming aware of the event<br><br>7 calendar days Only reportable if the event may have led to an SAE if;<br>• suitable action had not taken<br>• intervention had not been made<br>• if circumstances had been less fortunate
Urgent Safety Measures	By phone and email:<br><br>Substantial amendment form giving notice in writing setting out the reasons for the urgent safety measures and the plan for future action.	Chief Investigator	Main REC and Sponsor	Contact the Sponsor and MREC Immediately<br><br>Within 3 days<br><br>Main REC with a copy also sent to the sponsor. The MREC will acknowledge this within 30 days of receipt.
<u>Progress Reports</u>	Annual Progress Report Form (non-CTIMPs) available from the NRES website	Chief Investigator	Main REC with a copy to be sent to the Sponsor	Annually (starting 12 months after the date of favourable opinion)
<u>Declaration of the conclusion or early termination of the study</u>	End of Study Declaration form available from the NRES website	Chief Investigator	Main REC with a copy to be sent to the sponsor	Within 90 days (conclusion)<br><br>Within 15 days (early termination)

				The end of study definition should be clearly defined in the protocol
<u>Summary of final Report</u>	No Standard Format However, the following Information should be included:- Where the study has met its objectives, the main findings and arrangements for publication or dissemination including feedback to participants	Chief Investigator	Main REC with a copy to be sent to the sponsor	Within one year of conclusion of the Research

20.3 *Appendix 3: SADE Report Form – serious adverse device effect*

1. Project identifiers:

Site:	Investigator name:
IRAS number:	
Project Title:	

2. Site details:

Reported by:	Print name:
Tel:	Email:

3. Participant identifiers:

Participant Study ID:	Sex:<br>M ☐ F ☐	Age:

4. Event/Effect Information:

Date of Onset: __ __/__ __ _/__ __ __ _ dd / mm / yyyy
Event/ Effect:
Description of Event/Effect: *Further information e.g. Lab Tests/Results, Signs & Symptoms related to Diagnosis*

5. Action Taken Regarding Study Device:

None ☐ Removed/ Discontinued ☐ Other (specify)☐ ______________________________
Details treatment given:

6. Serious Criteria *(tick all that apply)*

Death	☐
Life threatening illness or injury	☐
Hospitalisation or prolonged of hospitalisation	☐
Permanent impairment of body structure or body function	☐
Medical or surgical intervention required to prevent any of the	☐
Led to foetal distress, foetal death or congenital anomaly or birth defect	☐
Other (maybe protocol specific)	☐ Please specify:___________________

7. **Causality: (*Detail all possible and suspected causes*)**

8. **Relationship of the Event/Effect to the Device?**

Related ☐ Not Related ☐

9. **Event/Effect anticipated?**

Anticipated ☐ Unanticipated ☐

10. **Classification of SADE:**

Device Related	Device Related Anticipated Serious Adverse Device Effect - (ASADE)	☐
	Unanticipated Serious Adverse Device Effect - (USADE)	☐
Not related to device	Not related to device Serious Adverse Event – (SAE)	☐

11. **Investigational Medical Device Information – *Detail all devices*:**

**Name of Device**	**Start date of device utilisation**	**End date of device utilisation**
	__ __/__ __ __/__ __ __ __<br>dd / mmm / yyyy	__ __/__ __ __/__ __ __ __<br>dd / mmm / yyyy
	__ __/__ __ __/__ __ __ __<br>dd / mmm / yyyy	__ __/__ __ __/__ __ __ __<br>dd / mmm / yyyy

12. **Event Outcome:**

1)	Recovered:	Yes: ☐ No: ☐	If yes, date of recovery:<br>__ __/__ __ __/__ __ __ __
2)	Ongoing:	Yes: ☐ No: ☐	If yes, details:
3)	Recovered with sequelae:	Yes: ☐ No: ☐	If yes, date:<br>__ __/__ __ __/__ __ __ __<br>Details:
4)	Subject Died:	Yes: ☐ No: ☐	If yes, date of death:<br>__ __/__ __ __/__ __ __ __

13. **Principal Investigator**

Name	Investigator Signature	Date:<br>__ __/__ __ __/__ __ __ __<br>dd / mmm / yyyy
PI Tel:		
PI Email:		

REPORTING INSTRUCTIONS

Send Copy to R&D <u>within 24hrs</u> from becoming aware of the event by either:

Email: R&D@gstt.nhs.uk
Fax: 0207 188 3472

Retain this form in the Trial Master File.

14. For Internal use (Chief Investigator)

CI Name:		
Confirmation of classification:	Confirm Classification: Yes ☐ No ☐ ⇨if no state reason and re-classify:	
CI Signature		Date: __ __/__ __ __/__ __ __ __ dd / mmm / yyyy
Action taken by Sponsor:		
Sponsor Signature		Date: __ __/__ __ __/__ __ __ __ dd / mmm / yyyy
Trust's Medical Devices Safety Officer Signature		Date: __ __/__ __ __/__ __ __ __ dd / mmm / yyyy

21 REFERENCES

1. Royal College of Physicians (2022) Falls and Fragility Fracture Audit Programme, National Hip Fracture Database Extended Report. https://www.nhfd.co.uk/2022report
2. Sheehan KJ, Williamson L, Alexander J, Filliter C, Sobolev B, Guy P, Bearne LM, Sackley C. Prognostic factors of functional outcome after hip fracture surgery: a systematic review. Age and ageing. 2018 Sep 1;47(5):661-70.
3. Dreinhofer KE, Mitchell PJ, Begue T, et al. A global call to action to improve the care of people with fragility fractures. *Injury* 2018;49(8):1393-97. doi: 10.1016/j.injury.2018.06.032
4. Handoll HH, Cameron ID, Mak JC, et al. Multidisciplinary rehabilitation for older people with hip fractures. *Cochrane Database Syst Rev* 2021;11:CD007125. doi: 10.1002/14651858.CD007125.pub3
5. National Clinical Guideline Centre. The management of hip fracture in adults. London: National Clinical Guidelines Centre, 2019. www.ncgc.ac.uk.
6. Resnick B, Beaupre L, McGilton KS, et al. Rehabilitation Interventions for Older Individuals With Cognitive Impairment Post-Hip Fracture: A Systematic Review. *J Am Med Dir Assoc* 2016;17(3):200-5. doi: 10.1016/j.jamda.2015.10.004
7. Pekkarinen T, Loyttyniemi E, Valimaki M. Hip fracture prevention with a multifactorial educational program in elderly community-dwelling Finnish women. *Osteoporos Int* 2013;24(12):2983-92. doi: 10.1007/s00198-013-2381-y
8. Edgren J, Rantanen T, Heinonen A, et al. Effects of progressive resistance training on physical disability among older community-dwelling people with history of hip fracture. *Aging Clin Exp Res* 2012;24(2):171-5.
9. National Health Service England. Next steps for risk stratification in the NHS. https://www.england.nhs.uk/wp-content/uploads/2015/01/nxt-steps-risk-strat-glewis.pdf, 2015.
10. Langford D, Edwards N, Gray SM, et al. "Life Goes On." Everyday Tasks, Coping Self-Efficacy, and Independence: Exploring Older Adults' Recovery From Hip Fracture. *Qual Health Res* 2018:1049732318755675. doi: 10.1177/1049732318755675
11. House of Lords Scientific and Technology Committee. Genomic medicine. Volume II: evidence. Stationery Office. 2009
12. Goubar A, Martin FC, Sackley C, et al. Development and validation of multivariable prediction models for in-hospital death, 30-day death, and change in residence after hip fracture surgery and the 'stratify-hip' algorithm. *J Gerontol A Biol Sci Med Sci* 2023 doi: 10.1093/gerona/glad053
13. Southwell J, Potter C, Wyatt D, Sadler E, Sheehan KJ. Older adults' perceptions of early rehabilitation and recovery after hip fracture surgery: a UK qualitative study. Disability and Rehabilitation. 2022 Mar 13;44(6):939-46.
14. Volkmer B, Sadler E, Lambe K, et al. Orthopaedic physiotherapists' perceptions of mechanisms for observed variation in the implementation of physiotherapy practices in the early postoperative phase after hip fracture: a UK qualitative study. *Age Ageing* 2021;50(6):1961-70. doi: 10.1093/ageing/afab131
15. Guerra S, Lambe K, Manolova G, Sadler E, Sheehan KJ. Multidisciplinary team healthcare professionals' perceptions of current and optimal acute rehabilitation, a hip fracture example A UK qualitative interview study informed by the Theoretical Domains Framework. Plos One. 2022 Nov 18;17(11):e0277986.
16. Lambe K, Guerra S, Salazar de Pablo G, Ayis S, Cameron ID, Foster NE, Godfrey E, Gregson CL, Martin FC, Sackley C, Walsh N. Effect of inpatient rehabilitation treatment ingredients on functioning, quality of life, length of stay, discharge destination, and mortality among older

adults with unplanned admission: an overview review. BMC geriatrics. 2022 Jun 11;22(1):501.
17. Diong J, Allen N, Sherrington C. Structured exercise improves mobility after hip fracture: a meta-analysis with meta-regression. *Br J Sports Med* 2016;50(6):346-55. doi: 10.1136/bjsports-2014-094465
18. Taylor NF, Harding KE, Dennett AM, et al. Behaviour change interventions to increase physical activity in hospitalised patients: a systematic review, meta-analysis and meta-regression. *Age Ageing* 2021 doi: 10.1093/ageing/afab154
19. Handoll HH, Sherrington C, Mak JC. Interventions for improving mobility after hip fracture surgery in adults. *Cochrane Database Syst Rev* 2011(3):CD001704. doi: 10.1002/14651858.CD001704.pub4
20. Beer N, Riffat A, Volkmer B, et al. Patient perspectives of recovery after hip fracture: a systematic review and qualitative synthesis. *Disabil Rehabil* 2021:1-16. doi: 10.1080/09638288.2021.1965228
21. Royal College of Physicians (2017) Falls and Fragility Fracture Audit Programme. Recovering after a hip fracture: helping people understand physiotherapy in the NHS. Physiotherapy 'Hip Sprint' audit report. https://www.rcplondon.ac.uk/projects/outputs/recovering-after-hip-fracture-helping-people-understand-physiotherapy-nhs.
22. McDonough CM, Harris-Hayes M, Kristensen MT, et al. Physical Therapy Management of Older Adults With Hip Fracture. *J Orthop Sports Phys Ther* 2021;51(2):CPG1-CPG81. doi: 10.2519/jospt.2021.0301
23. Chehade, M., and A. Taylor. "Australian and New Zealand guideline for hip fracture care-improving outcomes in hip fracture management of adults." (2014).
24. Jones J, Hunter D. Consensus methods for medical and health services research. *BMJ* 1995;311(7001):376-80. doi: 10.1136/bmj.311.7001.376
25. UK Standards for Public Involvement in Research 2016 https://sites.google.com/nihr.ac.uk/pi-standards/home.
26. Williams NH, Roberts JL, Din NU, et al. Fracture in the Elderly Multidisciplinary Rehabilitation (FEMuR): a phase II randomised feasibility study of a multidisciplinary rehabilitation package following hip fracture. *BMJ Open* 2016;6(10):e012422. doi: 10.1136/bmjopen-2016-012422
27. Sheehan KJ, Fitzgerald L, Hatherley S, et al. Inequity in rehabilitation interventions after hip fracture: a systematic review. *Age Ageing* 2019;48(4):489-97. doi: 10.1093/ageing/afz031
28. Royal College of Physicians (2019) Falls and Fragility Fracture Audit Programme, National Hip Fracture Database Extended Report
https://www.nhfd.co.uk/files/2019ReportFiles/NHFD_2019_Annual_Report_v101.pdf
29. Royal College of Physicians (2020) Falls and Fragility Fracture Audit Programme, National Hip Fracture Database Extended Report
https://www.nhfd.co.uk/20/hipfractureR.nsf/docs/reports2020
30. Hammond SP, Cross JL, Shepstone L, et al. PERFECTED enhanced recovery (PERFECT-ER) care versus standard acute care for patients admitted to acute settings with hip fracture identified as experiencing confusion: study protocol for a feasibility cluster randomized controlled trial. *Trials* 2017;18(1):583. doi: 10.1186/s13063-017-2303-y
31. Mental Capacity Act. http://www.legislation.gov.uk/ukpga/2005/9/contents, 2005.
32. Haywood KL, Griffin XL, Achten J, et al. Developing a core outcome set for hip fracture trials. *Bone Joint J* 2014;96-B(8):1016-23. doi: 10.1302/0301-620X.96B8.33766
33. Janssen MF, Pickard AS, Golicki D, et al. Measurement properties of the EQ-5D-5L compared to the EQ-5D-3L across eight patient groups: a multi-country study. *Qual Life Res* 2013;22(7):1717-27. doi: 10.1007/s11136-012-0322-4
34. Mahoney FI, Barthel DW. Functional Evaluation: The Barthel Index. *Md State Med J* 1965;14:61-5.

35. Harwood RH, Ebrahim S. The validity, reliability and responsiveness of the Nottingham Extended Activities of Daily Living scale in patients undergoing total hip replacement. *Disabil Rehabil* 2002;24(7):371-7. doi: 10.1080/10.1080/09638280110101541
36. Yardley L, Beyer N, Hauer K, et al. Development and initial validation of the Falls Efficacy Scale-International (FES-I). *Age Ageing* 2005;34(6):614-9. doi: 10.1093/ageing/afi196
37. Kempen GI, Yardley L, van Haastregt JC, et al. The Short FES-I: a shortened version of the falls efficacy scale-international to assess fear of falling. *Age Ageing* 2008;37(1):45-50. doi: 10.1093/ageing/afm157
38. Resnick B, Jenkins LS. Testing the reliability and validity of the Self-Efficacy for Exercise scale. *Nurs Res* 2000;49(3):154-9. doi: 10.1097/00006199-200005000-00007
39. Haefeli M, Elfering A. Pain assessment. *Eur Spine J* 2006;15 Suppl 1(Suppl 1):S17-24. doi: 10.1007/s00586-005-1044-x
40. Kristensen MT, Foss NB, Ekdahl C, et al. Prefracture functional level evaluated by the New Mobility Score predicts in-hospital outcome after hip fracture surgery. *Acta Orthop* 2010;81(3):296-302. doi: 10.3109/17453674.2010.487240
41. Baker PS, Bodner EV, Allman RM. Measuring life-space mobility in community-dwelling older adults. *J Am Geriatr Soc* 2003;51(11):1610-4. doi: 10.1046/j.1532-5415.2003.51512.x
42. Saunders B, Sim J, Kingstone T, et al. Saturation in qualitative research: exploring its conceptualization and operationalization. *Qual Quant* 2018;52(4):1893-907. doi: 10.1007/s11135-017-0574-8
43. Lorig KR, Sobel DS, Ritter PL, et al. Effect of a self-management program on patients with chronic disease. *Eff Clin Pract* 2001;4(6):256-62.
44. Michie S, van Stralen MM, West R. The behaviour change wheel: a new method for characterising and designing behaviour change interventions. *Implement Sci* 2011;6:42. doi: 10.1186/1748-5908-6-42
45. Hauer K, Rost B, Rutschle K, et al. Exercise training for rehabilitation and secondary prevention of falls in geriatric patients with a history of injurious falls. *J Am Geriatr Soc* 2001;49(1):10-20. doi: 10.1046/j.1532-5415.2001.49004.x
46. National Institute for Health and Care Excellence. Falls in older people: assessing risk and prevention. NICE; 2013.
47. Bonin-Guillaume S, Jouve E, Lauretta R, et al. Algoplus performance to detect pain in depressed and/or demented old patients. *Eur J Pain* 2016;20(7):1185-93. doi: 10.1002/ejp.844
48. Park SH, Cho YS. Predictive validity of the Cornell Scale for depression in dementia among older adults with and without dementia: A systematic review and meta-analysis. *Psychiatry Res* 2022;310:114445. doi: 10.1016/j.psychres.2022.114445
49. Eldridge SM, Chan CL, Campbell MJ, et al. CONSORT 2010 statement: extension to randomised pilot and feasibility trials. *Pilot Feasibility Stud* 2016;2:64. doi: 10.1186/s40814-016-0105-8
50. Detry MA, Lewis RJ. The intention-to-treat principle: how to assess the true effect of choosing a medical treatment. *JAMA* 2014;312(1):85-6. doi: 10.1001/jama.2014.7523
51. Braun V, Clarke V. Using thematic analysis in psychology. *Qualitative research in psychology* 2006;3(2):77-101.
52. Atkins L, Francis J, Islam R, et al. A guide to using the Theoretical Domains Framework of behaviour change to investigate implementation problems. *Implement Sci* 2017;12(1):77. doi: 10.1186/s13012-017-0605-9
53. Avery KN, Williamson PR, Gamble C, et al. Informing efficient randomised controlled trials: exploration of challenges in developing progression criteria for internal pilot studies. *BMJ Open* 2017;7(2):e013537. doi: 10.1136/bmjopen-2016-013537
