# Supplementary material for: Structured tailored rehabilitation after hip fragility fracture: The ‘Stratify’ feasibility and pilot randomised controlled trial protocol
Source: PLoS One. 2024 Dec 17;19(12):e0306870. doi: 10.1371/journal.pone.0306870 (PMC11651604; doi:10.1371/journal.pone.0306870)
Supplement: S3 File — (PDF) [file pone.0306870.s003.pdf]

PARTICIPANT CONSENT FORM

IRAS ID: 312631

Study Number: _______________

Participant Identification Number for this trial: ___________

Title of Project: Structured Tailored Rehabilitation After Hip Fragility Fracture: The 'STRATIFY' Feasibility Randomised Controlled Trial.

Name of Researcher: Julie Whitney

Please initial box

1. I confirm that I have read the information sheet dated................... (version...........) for the above study. I have had the opportunity to consider the information, ask questions and have had these answered satisfactorily. ☐
2. I understand that my participation is voluntary and that I am free to withdraw at any time without giving any reason, without my medical care or legal rights being affected. ☐
3. I give permission for the Kings College London research team to access my medical records for the purposes of this research study. ☐
4. I understand that relevant sections of my medical notes and data collected during the study, may be looked at by individuals from the Sponsor (Guy's and St Thomas' NHS Foundation Trust and King's College London), from regulatory authorities or from the NHS Trust, where it is relevant to my taking part in this research. I give permission for these individuals to have access to my data and/or medical records. ☐
5. I understand that information about me collected during the study, may be passed onto the community team who will be looking after me when I go home. ☐
6. I give permission for my personal information (including name, address, phone number and consent form) to be passed to King's College London for administration of the study. ☐
7. I understand that the information held and maintained by Guys and St Thomas NHS Foundation Trust may be used to help contact me or provide information about my health status. ☐
8. I understand that data collected about me during the study will be converted to anonymised data and stored indefinitely in an open access repository - the King's Open Research Data System for future ethically approved research studies. ☐
9. I understand that identifiable data collected about me during the study will be stored securely for 5 years after which it will be destroyed in line with Guys and St Thomas NHS Foundation Trust/King's College London policies on data destruction. ☐
10. I agree to be contacted by phone/post by the research team to complete questionnaires. ☐
11. I understand that I can contact the research team during/after the study to seek information on the results of the study. ☐

Yes ☐ ☐

When completed: 1 for participant; 1 for researcher site file; 1 to be kept in medical notes.

12. I agree to be contacted by phone by the research team for an interview about my experience of being in the study, and for this to be audio recorded, and for the audio recording to be transcribed by an external company (optional). No

13. I would like to receive a lay summary by post that summarises findings after the study ends (optional). Yes ☐ No ☐

14. I agree to take part in the above study Yes ☐ No ☐

______________________ ______________________ ______________________
Name of Participant Date Signature

______________________ ______________________ ______________________
Name of person taking consent Date Signature

Preferred contact details for sharing results of the study (if applicable)

____

____

____

____

____

When completed: 1 for participant; 1 for researcher site file; 1 to be kept in medical notes.
