# Supplementary material for: Structured tailored rehabilitation after hip fragility fracture: The ‘Stratify’ feasibility and pilot randomised controlled trial protocol
Source: PLoS One. 2024 Dec 17;19(12):e0306870. doi: 10.1371/journal.pone.0306870 (PMC11651604; doi:10.1371/journal.pone.0306870)
Supplement: S5 File — (PDF) [file pone.0306870.s005.pdf]

IRAS ID: 312631

Study Number:

Participant Identification Number for this trial:

CONSULTEE DECLARATION FORM

Title of Project: **Structured Tailored Rehabilitation After Hip Fragility Fracture: The 'STRATIFY' Feasibility Randomised Controlled Trial**

Name of Researcher: Julie Whitney

Please initial box

1. I, …………………., confirm that I have been consulted about …………………………. participation in this research project. I have had the opportunity to consider the information, ask questions and have had these answered satisfactorily. ☐
2. In my opinion the person who I am supporting would have no objection to taking part in the above study. ☐
3. I understand that I can request the person who I am supporting to be withdrawn from the study at any time, without giving any reason and without their care or legal rights being affected. ☐
4. I give permission for the King's College London research team to access the person who I am supporting's medical records for the purposes of this research study. ☐
5. I understand that relevant sections of the person who I am supporting's medical notes and data collected during the study, may be looked at by individuals from the Sponsor (Guy's and St Thomas' NHS Foundation Trust and King's College London), from regulatory authorities or from the NHS Trust, where it is relevant to the person who I am supporting's involvement in this research. I give permission for these individuals to have access to the person who I am supporting 's data and/or medical records. ☐
6. I understand that information about the person who I am supporting that is collected during the study, may be passed onto the community team who will be looking after them when they go home. ☐
7. (If appropriate) I give permission for my own and the person who I am supporting's personal information (including name, address, phone number, and consent form) to be passed to King's College London for administration of the study. ☐
8. I understand that data collected about the person who I am supporting during the study will be converted to anonymised data and stored indefinitely in an open access repository - the King's Open Research Data System for future ethically approved research studies. ☐
9. I understand that identifiable data collected about me and the person who I am supporting during the study will be stored securely for 5 years after which it will be destroyed in line with Guys and St Thomas NHS Foundation Trust/King's College London policies on data destruction. ☐
10. I understand the King's College London research team may approach the carer of the person I support to invite them to take part in the study. ☐
11. I understand that I can contact the research team during/after the study to seek information on the results of the study. ☐

When completed: 1 for participant; 1 for researcher site file; 1 to be kept in medical notes.

12. I agree to support the completion of questionnaires about the person who I am supporting (optional). Yes ☐ No ☐

13. I agree to provide my contact details so that the research team can follow up by phone to complete questionnaires (optional). Yes ☐ No ☐

14. I would like the Kings College London research team to send me a summary of the study findings by post after the study ends (optional). Yes ☐ No ☐

15. I would like the Kings College London research team to send the person who I am supporting a summary of the study findings by post after the study ends (optional). Yes ☐ No ☐

____________________ ____________________________

Name of Consultee Relationship to participant:

____________________ ____________________

Date Signature

___________________________ ____________________ ____________________

Person obtaining declaration Date Signature

Preferred contact details for sharing results of the study with me (if applicable)

____

____

____

Preferred contact details for sharing results of the study with the person who I am supporting (if applicable)

____

____

____
