# Supplementary material for: Structured tailored rehabilitation after hip fragility fracture: The ‘Stratify’ feasibility and pilot randomised controlled trial protocol
Source: PLoS One. 2024 Dec 17;19(12):e0306870. doi: 10.1371/journal.pone.0306870 (PMC11651604; doi:10.1371/journal.pone.0306870)
Supplement: S7 File — (PDF) [file pone.0306870.s007.pdf]

STRATIFY HIP

Structured **T**ailored **R**ehabilitation **A**f**T**er h**I**p **F**ragilit**Y** fracture

Virtual Nominal Group Technique
e-Booklet
May 2022

For correspondence:
Dr Katie Sheehan
Katie.Sheehan@kcl.ac.uk

TABLE OF CONTENTS

Purpose 3
Context 3
  Target population 3
  Target setting 3
  Target outcomes 3
Developing a stratified approach 4
  Defining subgroups 4
  Characteristics of subgroups 5
  Matching subgroups to interventions tailored to their needs 5
Nominal group technique process 6
  Questions 6
Intervention theoretical framework 7
Patient and carer perspective 8
  Public and patient involvement 8
  Evidence from qualitative synthesis (patient perspective) 9
  Evidence from qualitative synthesis (carer perspective) 9
Evidence for inpatient rehabilitation 10
  Hip fracture guidelines 10
  Hip fracture systematic reviews 12
  Older adults 13
References 15

PURPOSE

The purpose of this intervention development workshop is to generate a set of treatment components to inform programme theories and a logic model for a future intervention of stratified acute rehabilitation (led by physiotherapy and occupational therapy) after hip fracture. The intervention will include physiotherapy and occupational therapy tailored to the needs of patients in each of three risk strata.

The structure of this workshop and its associated materials were informed by previous successful remote intervention development workshops for rehabilitation after stroke.[1]

CONTEXT

Target population

The target population for the intervention is patients with hip fracture.

Each year, United Kingdom (UK) hospitals admit over 65,000 men and women aged 60 years or more with hip fracture.[2] The average patient with hip fracture is 84 years old, frail, and has at least one comorbidity (one quarter present with a dementia diagnosis).[3] Most were living at home and able to walk outdoors before their fracture.[3]

Even with surgery, 7% of patients die within 30-days.[3] Among survivors, 22% transition from independent living to care homes.[4]

Target setting

The intervention will start after surgery and end when a patient is discharged from the acute ward. The intervention needs to be feasibility for delivery within the NHS given available resource and average acute ward length of stay (15.2 days for 2019 i.e. pre-Covid).[5]

Target outcomes

We will capture outcomes recommended by the Haywood Core Outcome Set (COS) for hip fracture trials namely mortality, pain (visual analogue scale), activities of daily living (Barthel Index), mobility (cumulated ambulation score - (getting in and out of bed, sit-to-standto-sit from a chair, and walking), and health-related quality of life (EuroQoL EQ-5D-5L).[6]

We will also capture discharge destination as a key outcome for defining risk strata.

DEVELOPING A STRATIFIED APPROACH

Stratified rehabilitation considers an entire population competing for resources to identify subgroups of patients with different risk of poor outcomes.[7] Subgroups are then matched to rehabilitation tailored to their needs to optimise outcomes across the entire population.

Hip fracture survivors recently described this tailored approach as key to successful recovery.[8] Further, a stratified approach is regarded central to the progress of healthcare according to the NHS[7] and House of Lords Science and Technology Committee.[9]

Defining subgroups

We developed and validated an approach to identify patients at low-, intermediate- and high-risk using records for over 170,000 patients admitted to one of 173 hospitals in England and Wales.[10]

High: high risk of inhospital death, high or medium risk of 30-day death

Medium: medium or high risk of change in residence

Low: low risk of death or change in residence (to higher level of care)

The approach requires website entry of 5 pieces of information (age, sex, prefracture mobility, prefracture residence, and dementia diagnosis) to generate a risk assignment (Figure 1).

Figure 1: Screenshot of website for identification of subgroups with differing risk of poor outcome. Example displayed indicates for a woman aged 60-64 years, who was previously able to walk indoors and outdoors, was admitted from home without dementia, the risk of a poor outcome is low -for each outcome and for overall.

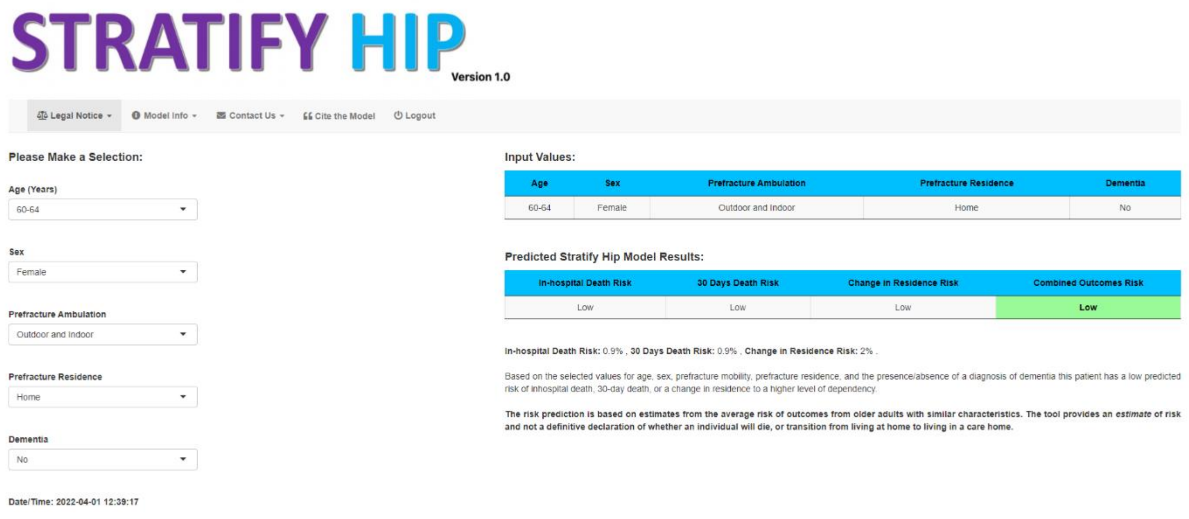

Characteristics of subgroups

40% of patients fall into the **high-risk category**. Most of these patients are over 80 years old, include both women and men, and were not able to walk outdoors before their fracture. Almost half were admitted from a care home and/or with a diagnosis of dementia.

40% of patients fall into the **medium-risk category**. Most of these patients are over 80 years old, almost all are women, admitted from home, and able to walk outdoors before their fracture. One in ten have a dementia diagnosis.

20% of patients fall into the **low-risk category**. These patients are the youngest (all between 60 and 80 years old), include both men and women, without a diagnosis of dementia, admitted from home, and able to walk outdoors before their fracture.

Matching subgroups to interventions tailored to their needs

The next step in developing a stratified approach to care is to design interventions for each subgroup tailored to their needs. We will use an evidence-informed nominal group technique described in the next section to inform interventions.

NOMINAL GROUP TECHNIQUE PROCESS

Nominal Group Technique is a structured method to reach consensus in small group discussions.[11] The approach has been successfully modified for remote delivery.[1]

A group of experts meet to individually provide ideas for pre-determined questions, which are then shared, clarifications sought, prior to prioritization via scoring by all group members. More specific detail of each step is detailed in Figure 2.

Figure 2: Nominal group technique

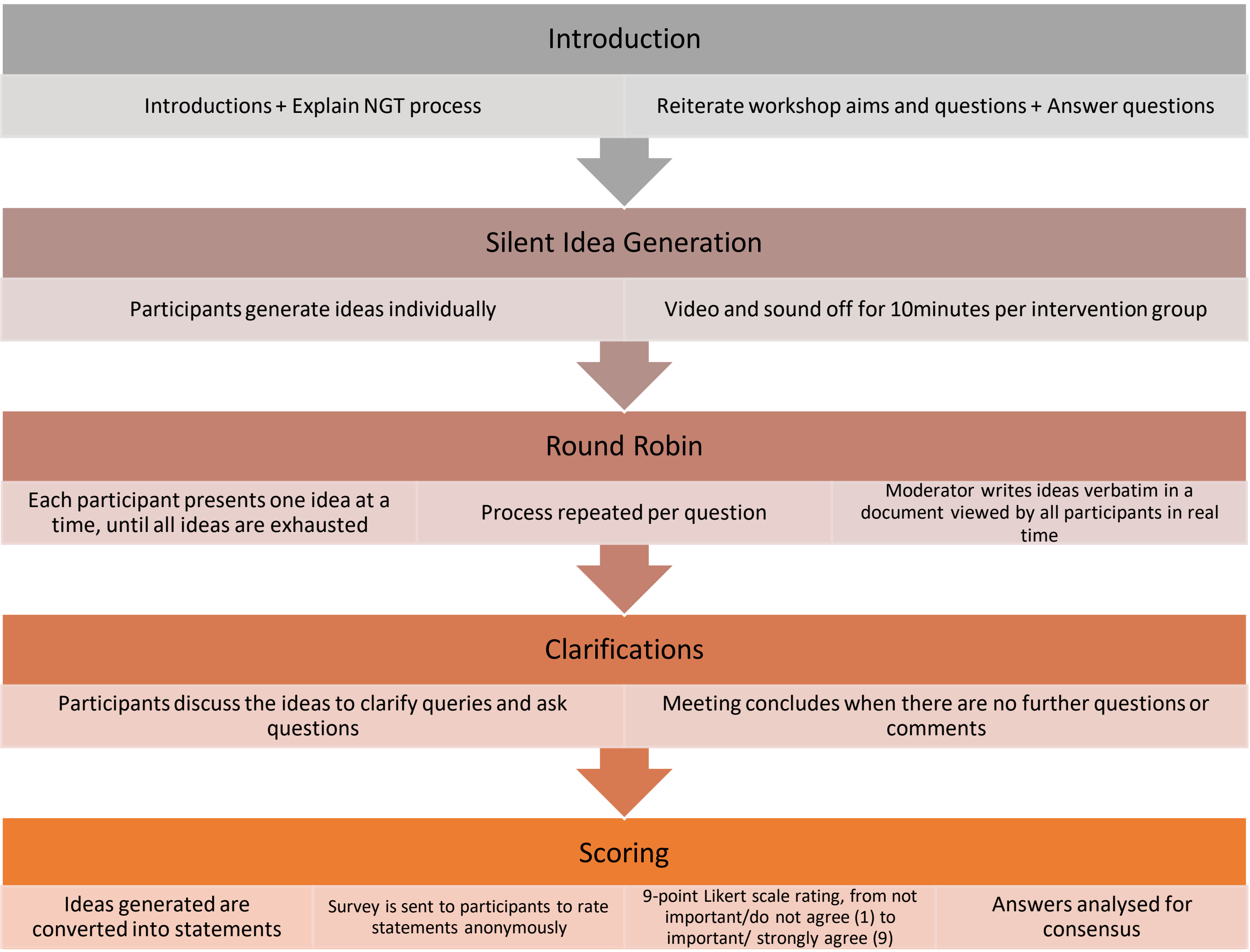

Interventions for subgroups– Nominal Group Technique Question

What treatment components should be included in physiotherapy and occupational therapy management to optimize outcomes of inpatient rehabilitation for patients in the:

1. low-risk group,
2. medium-risk group, and
3. high-risk group?

INTERVENTION THEORETICAL FRAMEWORK

Normalisation Process Theory[12] is a theoretical framework from implementation science which seeks to embed a practice into 'work as usual' through four components:

1. Coherence relates to understanding and making sense of a practice (here 'stratified approach to care')
2. cognitive participation – engagement of physiotherapists and occupational therapists with the practice
3. collective action – the joint 'work' of physiotherapists, occupational therapists, patients (and carers) needed to enact the practice
4. reflexive monitoring – physiotherapist and occupational therapist reflection and appraisal of the practice over time to ensure it becomes routinely embedded

This theory was considered essential for designing an intervention with future sustainability in mind.

PATIENT AND CARER PERSPECTIVE

Public and patient involvement

TROOP [Trauma Rehabilitation (Orthopaedic) for Older People] is a public and patient involvement group facilitated by the research team at Kings College London. The group of patients and carers meets at least four times per year to inform the design, delivery, and dissemination of research in the field. TROOP met in April 2022 where they discussed what was important during early in-hospital rehabilitation after hip fracture surgery. Their views were captured and visually presented in a word cloud (Figure 3).

Figure 3: Word cloud of patient and carer perspectives on important aspects of early in-hospital rehabilitation after hip fracture surgery.

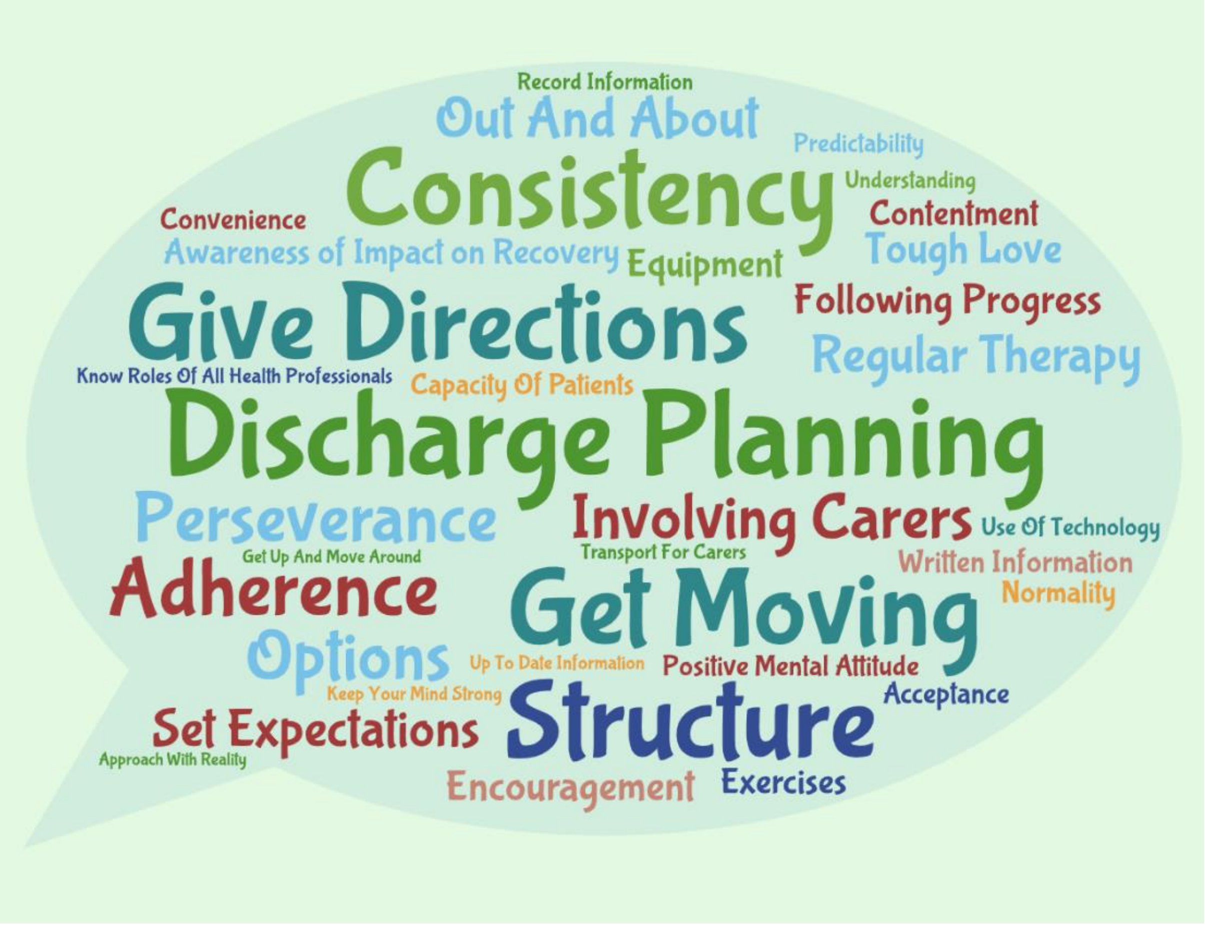

Evidence from qualitative synthesis (patient perspective)

We synthesised the evidence from 14 studies which explored 279 patient perspectives of recovery after hip fracture.[13]

Across studies, patients considered recovery as a return to prefracture activities or ‘normal’ enabling independence.[13] These activities were often described in terms of mobility such as activities of daily living, walking, and participating in social events. Participants in some studies indicated they were willing to accept a new normal if it enabled a sense of identity or preserved abilities to complete activities albeit in a different manner. These priorities reflect the World Health Organization’s definition of functional ability as ‘all the health-related attributes that enable people to be and to do what they have reason to value’.[14]

Additional themes relevant to the early postoperative phase noted in the review included:

1. New anxieties related to fear of falling, ability to cope on discharge home, and feasibility of returning to valued activities. These were particularly evident among the oldest participants and those with poorer mobility/other health conditions.
2. A need to engage with and drive recovery. This was considered reliant on a clear understanding of the process with realistic expectations and goals. It was acknowledged this may be difficult for some patients e.g. those with cognitive impairment.
3. A reliance on professional support which was particularly strong in the early postoperative phase where participants reported wanting more (earlier and more regular) rehabilitation during this stage to support their recovery.
4. A reliance on social support to enable participants to be discharged home from hospital. This was accompanied by feelings of guilt related to new levels of dependency and mitigated by the preparation of mobility aids and changes in the home.

Evidence from qualitative synthesis (carer perspective)

Saletti-Cuesta and colleagues synthesized the evidence from 21 studies which explored 318 informal carers’ experiences of caring for older people with a hip fracture.[15] Most carers were women and spouses or children of a person with hip fracture.

Key implications from the work:

1. Including carers in umbrella of care within a carer-centred approach
2. Involving carers within shared decision-making about care requirements and rehabilitation goals
3. Providing experiential learning to help develop skills in relation to carer roles
4. Providing opportunities for carers to explore ways of sustaining their own health through self-compassion

This led 81 global societies to endorse a call to action to improve acute multidisciplinary care after hip fracture.[16]

Hip fracture guidelines

UK

National Institute for Health and Care Excellence guidance[17]

Items that relate to physiotherapy/occupational therapy:

- offer physiotherapy assessment and unless medically or surgically contraindicated, mobilisation on the day after surgery
- offer mobilisation at least once a day and ensure regular physiotherapy review
- early identification of individual goals for multidisciplinary rehabilitation to recover mobility and independence, and to facilitate return to pre-fracture residence and long-term wellbeing
- continued, coordinated, orthogeriatric and multidisciplinary review
- liaison or integration with related services, particularly mental health, falls prevention, bone health, primary care and social services
- clinical and service governance responsibility for all stages of the pathway of care and rehabilitation
- consider early supported discharge if has capacity to participate in rehabilitation, able to transfer and mobilise short distances, and has yet to achieve full rehabilitation potential (inclusive of those from care homes)
- offer patients and/or carers verbal and printed information about treatment and care including rehabilitation programme, long-term outcomes, health professionals involved

The Chartered Society of Physiotherapy[18]

- A physiotherapist assesses all patients on the day of, or day following hip fracture surgery
- All patients are mobilised on the day of, or day following hip fracture surgery
- All patients receive daily physiotherapy that should total at least two hours in the first 7 days post-surgery
- All patients receive at least two hours of rehabilitation in subsequent weeks post-surgery until they have achieved their goals

USA

American Physical Therapy Association Clinical Practice Guideline [19]

Strong-moderate evidence

- Participate in nonpharmacological intervention programmes delivered by an interprofessional team to prevent delirium
- Assess and document risk factors for falls and contribute to interprofessional management

- Provide structured exercise inclusive of high-intensity resistive strength, balance, weight bearing and functional mobility training irrespective of dementia status
- Management should be in a multidisciplinary orthogeriatric program inclusive of physical therapy and early mobilisation
- Daily physiotherapy with duration as tolerated including instruction in a home programme
- Transfer from bed and ambulation should be provided as soon as possible after surgery and at least daily thereafter, unless contraindicated for medical/surgical reasons

Weak evidence

- Upper-body aerobic training
- Electrical stimulation for quadriceps strengthening and/or pain

Australia and New Zealand

Australian and New Zealand Guideline for Hip Fracture Care[20]

- Mobilisation should start on the day after surgery, unless medically or surgically contraindicated
- Offer patients a physiotherapy assessment on the day after surgery
- Offer patients mobilisation at least once a day and ensure regular physiotherapy review
- early identification of individual goals for multidisciplinary rehabilitation to recover mobility and independence, and to facilitate return to pre-fracture residence and long-term wellbeing
- early identification of most appropriate service to deliver rehabilitation
- continued, coordinated, orthogeriatric and multidisciplinary review and discharge planning liaison or integration with related services, including falls prevention, secondary fracture prevention, mental health, cultural services, primary care, community support services and carer support services.

Canada

Canadian National Hip Fracture Toolkit

- mobilised as soon as medically stable, progressing to standing within 24 hours of surgery with weight bearing status ‘as-tolerated’
- ambulation aids should be assessed, and their use taught, patients who are safe to
- ambulate with the aid independently should have access to the aid to promote independence
- at least daily physical and occupational therapy including weekends regardless of cognitive status
- physiotherapy should focus on gait quality, walking endurance, transfers, activities of daily living and safety
- occupational therapists provide support around, feeding/swallowing, positioning, skin protection, pain management and home preparation

- treatment goals (based on prefracture capacity) should progress ambulation, transfer, and activities of daily living
- intensity and frequency of treatment should be increased within patient tolerance and aligned with an activity plan focused on patient's needs in the home environment (mobilisation, stairs, elimination of trip and slip hazards, activities of daily living)
- all care staff should be involved in encouraging mobility/independence in toileting and transfers
- patients should be up in a chair for all meals and spend as much of the day as tolerated out of bed
- independence in self-care and hygiene should be encouraged with assistance as necessary
- coordination of rehabilitation and pain management, pain should be assessed regularly to ensure appropriately controlled
- daily assessment of progress to determine needs for postacute functional recovery and prevent delays in transitions in care
- discharge supported by all staff with early discussion with transfer facilities

Hip fracture systematic reviews

Handoll 2021: Multidisciplinary rehabilitation for older people with hip fractures

Data: 28 randomised controlled trials involving 5,351 participants. Most had unclear or high risk of bias in one or more aspect of their design.

Main findings:

- Moderate certainty evidence hospital multidisciplinary rehabilitation led by a medical specialist result in fewer cases of death or change in residence.
- Low certainty evidence hospital multidisciplinary rehabilitation reduces the number of people with poorer mobility at 12-months.
- Unclear which components of inpatient multidisciplinary rehabilitation optimise recovery in hospital (no between group differences for different models of multidisciplinary care), or of the effect on quality of life and activities of daily living.

Diong 2015: Structured exercise improves mobility after hip fracture: a meta-analysis with meta-regression[21]

Data: 19 moderate-high quality randomised controlled trials (13 in meta-analysis involving 1,903 participants). Setting included hospital (n = 10), hospital and community (n = 3), community (n = 6).

Main findings:

- Structured exercise led to small benefits in terms of overall mobility.
- Interventions which included progressive resistance exercise in particular saw greater benefits in terms of mobility. The potential for benefit from this type of exercise may only be for certain subgroups of patients (e.g. those who are weaker at baseline).

- Interventions that were delivered in settings other than hospital alone saw greater benefit for overall mobility. This may be related to intervention duration (longer for those who included settings other than hospital).
- No impact of a balance component, supervision, or dose of intervention on mobility. Possibly due to the small sample sizes in the meta-regression for these parameters.
- Most interventions were high dose -30 hours on average.

Handoll 2011: Interventions for improving mobility after hip fracture surgery in adults[22]

Data: 19 small and methodologically flawed randomised controlled trials (involving 1,589 participants). Setting included hospital (n = 12) and after discharge (n = 7).

Main findings:

- Insufficient and low-quality evidence to inform appropriate methods to improve mobility after hip fracture.
- For hospital-based interventions, there was inconsistent evidence for weight-bearing programmes, resistance training (including quadriceps strengthening), electrical stimulation, and early mobilisation.
- There was no evidence of benefit from treadmill training in hospital.
- Of two trials evaluating more intensive physiotherapy, one found no difference in recovery, the other reported a higher level of drop-out in the more intensive group.

Older adults

Lambe 2022: Treatment ingredients for inpatient rehabilitation[23]

Data: Systematic reviews (n = 12) of moderate to low quality, including 44 non-overlapping relevant RCTs which looked at the effectiveness of inpatient rehabilitation treatment ingredients (versus any comparison) on functioning, quality of life, length of stay, discharge destination, and mortality at intervention end among older adults with an unplanned hospital admission.

Main findings:

- When incorporated in a rehabilitation intervention, there was a large effect of *endurance exercise* (treadmill training, pedal/cycle ergometer, walking programme), *early intervention* (early start to physiotherapy, early discharge planning) and *shaping knowledge* (instructions on how to perform a behaviour in person/with leaflet) on walking endurance after the inpatient stay versus comparison.
- *Early intervention*, *repeated practice activities* (activity of daily living training, transfer practice), *goals and planning* (action planning, goal setting for behaviour or outcome), *increased medical care* (pain monitoring, oxygen, monitoring for complications) and/or *discharge planning* (early with multidisciplinary input) increased the likelihood of discharge home versus comparison.
- Evidence for other outcomes was inconclusive or no effect noted overall (and therefore the role of individual treatment ingredients was not explored).

Taylor 2021: Behaviour change interventions[24]

Data: Taylor and colleagues synthesised the evidence from 20 randomised controlled trials of behaviour change interventions on outcomes including physical activity levels, mobility, and length of stay in hospitalised patients (weighted mean age 66.7 years).

Main findings:

1. 23 behaviour change techniques with most trials using more than one technique. Most common included goal setting, feedback, reviewing behavioural goals, and instructions on how to perform a behaviour.
2. Moderate certainty evidence for small to moderate increases in physical activity levels following behaviour change interventions. Notably goal setting and feedback behaviour change techniques.
3. Inconclusive evidence from meta-analysis for mobility and length of stay.
4. Additional outcomes were assessed for few trials that could not be included in meta-analyses due to differences in the underlying population.

REFERENCES

1. Fisher RJ, Riley-Bennett F, Russell L, et al. Nominal group technique to establish the core components of home-based rehabilitation for survivors of stroke with severe disability. *BMJ Open* 2021;11(12):e052593. doi: 10.1136/bmjopen-2021-052593 [published Online First: 2021/12/04]
2. Royal College of Physicians. Falls and Fragility Fracture Audit Programme, National Hip Fracture Database Extended Report. 2014 http://wwwnhfdcouk/20/hipfractureRnsf/vwcontent/2014reportPDFs/$file/NHFD2014ExtendedReportpdf?OpenElement
3. Goubar A, Martin FC, Potter C, et al. The 30-day survival and recovery after hip fracture by timing of mobilization and dementia : a UK database study. *Bone Joint J* 2021;103-B(7):1317-24. doi: 10.1302/0301-620X.103B7.BJJ-2020-2349.R1
4. Sheehan KJ, Williamson L, Alexander J, Filliter C, Sobolev B, Guy P, Bearne LM, Sackley C. Prognostic factors of functional outcome after hip fracture surgery: a systematic review. Age and ageing. 2018 Sep 1;47(5):661-70.
5. Royal College of Physicians. National Hip Fracture Database Annual Report, 2020. https://www.nhfd.co.uk/FFFAP/Reports.nsf/0/220AC3A08F5AC22080258789007CCC92/$file/NHFD_2021_Report.pdf
6. Haywood KL, Griffin XL, Achten J, et al. Developing a core outcome set for hip fracture trials. *Bone Joint J* 2014;96-B(8):1016-23. doi: 10.1302/0301-620X.96B8.33766
7. National Health Service England. Next steps for risk stratification in the NHS. https://www.england.nhs.uk/wp-content/uploads/2015/01/nxt-steps-risk-strat-glewis.pdf, 2015.
8. Langford D, Edwards N, Gray SM, et al. "Life Goes On." Everyday Tasks, Coping Self-Efficacy, and Independence: Exploring Older Adults' Recovery From Hip Fracture. *Qual Health Res* 2018:1049732318755675. doi: 10.1177/1049732318755675
9. House of Lords Science and Technology Committee. Genomic medicine. Volume II: evidence. Stationery Office. 2009
10. Goubar A, Martin FC, Sackley C, et al. Development and validation of multivariable prediction models for inhospital death, 30-day death, and change in residence after hip fracture surgery: the 'Stratify-Hip' tool. *Journals of Gerontology Series A* 2022;Submitted.
11. Delbecq AL, Vandeven AH. Group Process Model for Problem Identification and Program Planning. *Journal of Applied Behavioral Science* 1971;7(4):466-&. doi: Doi 10.1177/002188637100700404
12. Murray E, Treweek S, Pope C, et al. Normalisation process theory: a framework for developing, evaluating and implementing complex interventions. *BMC Med* 2010;8:63. doi: 10.1186/1741-7015-8-63
13. Beer N, Riffat A, Volkmer B, Wyatt D, Lambe K, Sheehan KJ. Patient perspectives of recovery after hip fracture: a systematic review and qualitative synthesis. Disability and Rehabilitation. 2021 Sep 16:1-6.
14. World Health Organisation.. World Report on Ageing and Health. . In: WHO, ed. Geneva, Switzerland, 2016.
15. Saletti-Cuesta L, Tutton E, Langstaff D, et al. Understanding informal carers' experiences of caring for older people with a hip fracture: a systematic review of qualitative studies. *Disabil Rehabil* 2016:1-11. doi: 10.1080/09638288.2016.1262467

16. Dreinhofer KE, Mitchell PJ, Begue T, et al. A global call to action to improve the care of people with fragility fractures. *Injury* 2018;49(8):1393-97. doi: 10.1016/j.injury.2018.06.032
17. National Clinical Guideline Centre. The management of hip fracture in adults. . London: Royal College of Physicians (UK), 2017.
18. Royal College of Physicians. Recovering after a hip fracture: helping people understand physiotherapy in the NHS. Physiotherapy 'Hip Sprint' audit report. 2017. https://www.rcplondon.ac.uk/projects/outputs/recovering-after-hip-fracture-helping-people-understand-physiotherapy-nhs
19. McDonough CM, Harris-Hayes M, Kristensen MT, et al. Physical Therapy Management of Older Adults With Hip Fracture. *J Orthop Sports Phys Ther* 2021;51(2):CPG1-CPG81. doi: 10.2519/jospt.2021.0301.
20. Australian and New Zealand Guideline for Hip Fracture Care: Improving Outcomes in Hip Fracture Management of Adults. Sydney: Australian and New Zealand Hip Fracture Registry Steering Group; 2014.
21. Diong J, Allen N, Sherrington C. Structured exercise improves mobility after hip fracture: a meta-analysis with meta-regression. British journal of sports medicine. 2016 Mar 1;50(6):346-55.
22. Handoll HH, Sherrington C, Mak JC. Interventions for improving mobility after hip fracture surgery in adults. *Cochrane Database Syst Rev* 2011(3):CD001704. doi: 10.1002/14651858.CD001704.pub4
23. Lambe K GS, Salazar de Pablo G, Ayis S, Cameron ID, Foster NE, Godfrey E, Gregson CL, Martin FC, Sackley C, Walsh N, Sheehan KJ. Effect of inpatient rehabilitation treatment ingredients on functioning, quality of life, length of stay, discharge destination, and mortality among older aduls with unplanned admission: an overview review. *BMC Geriatrics* 2022; Revisions under review.
24. Taylor NF, Harding KE, Dennett AM, et al. Behaviour change interventions to increase physical activity in hospitalised patients: a systematic review, meta-analysis and meta-regression. *Age Ageing* 2021 doi: 10.1093/ageing/afab154.
