# Supplementary figures and images for: Structured tailored rehabilitation after hip fragility fracture: The ‘Stratify’ feasibility and pilot randomised controlled trial protocol
Source: PLoS One. 2024 Dec 17;19(12):e0306870. doi: 10.1371/journal.pone.0306870 (PMC11651604; doi:10.1371/journal.pone.0306870)

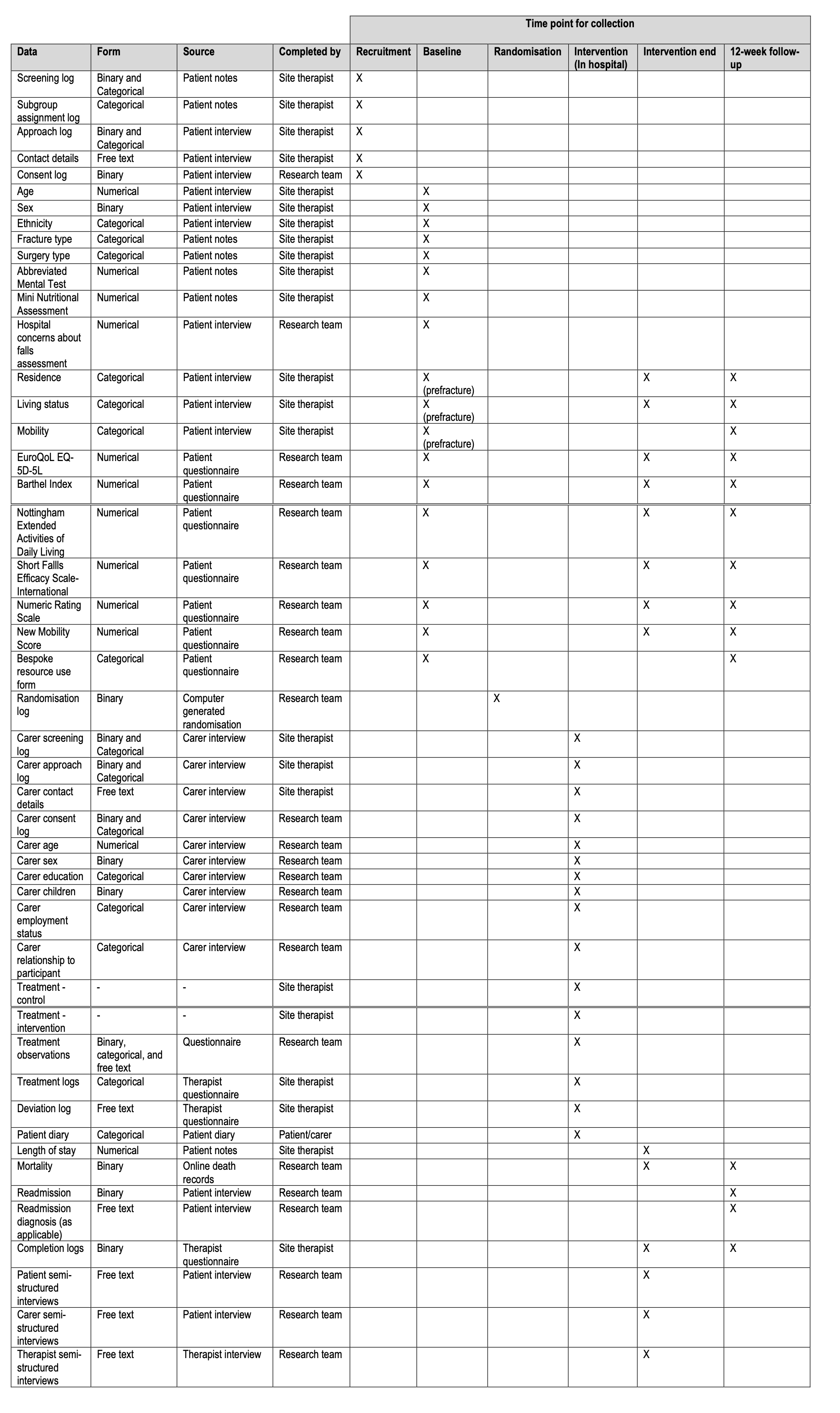

Supplement: S1 Table — (ZIP) [file pone.0306870.s008.zip › S3_Table.tiff]
